# Single-neuron mechanical perturbation evokes calcium plateaus that excite and modulate the network

**DOI:** 10.1038/s41598-023-47090-z

**Published:** 2023-11-24

**Authors:** Bogdana Cepkenovic, Florian Friedland, Erik Noetzel, Vanessa Maybeck, Andreas Offenhäusser

**Affiliations:** 1 Institute of Biological Information Processing: Bioelectronics (IBI-3), Forschungszentrum Jülich GmbH, Wilhelm-Johnen-Straße 1, 52428 Jülich, Germany; 2 Institute of Biological Information Processing: Mechanobiology (IBI-2), Forschungszentrum Jülich GmbH, Wilhelm-Johnen-Straße 1, 52428 Jülich, Germany; 3 RWTH Aachen University, Templergraben 55, 52062 Aachen, Germany

**Keywords:** Cellular neuroscience, Neural circuits, Intrinsic excitability, Action potential generation, Gap junctions, Intracellular recording, Patch clamp

## Abstract

Mechanical stimulation is a promising means to non-invasively excite and modulate neuronal networks with a high spatial resolution. Despite the thorough characterization of the initiation mechanism, whether or how mechanical responses disperse into non-target areas remains to be discovered. Our in vitro study demonstrates that a single-neuron deformation evokes responses that propagate to about a third of the untouched neighbors. The responses develop via calcium influx through mechanosensitive channels and regeneratively propagate through the neuronal ensemble via gap junctions. Although independent of action potentials and synapses, mechanical responses reliably evoke membrane depolarizations capable of inducing action potentials both in the target and neighbors. Finally, we show that mechanical stimulation transiently potentiates the responding assembly for further inputs, as both gain and excitability are transiently increased exclusively in neurons that respond to a neighbor’s mechanical stimulation. The findings indicate a biological component affecting the spatial resolution of mechanostimulation and point to a cross-talk in broad-network mechanical stimulations. Since giga-seal formation in patch-clamp produces a similar mechanical stimulus on the neuron, our findings inform which neuroscientific questions could be reliably tackled with patch-clamp and what recovery post-gigaseal formation is necessary.

## Introduction

Due to mechanosensitive (MS) channel expression, neurons respond promptly to acute membrane deformation^1–5^. Findings from mechanobiology have consistently demonstrated the neurons’ sensitivity to different mechanostimulation modalities^5,6^, along with the capacity to differentiate between different ranges of mechanical forces, from pN^7,8^, to nN^9^ and µN^10,11^. Accordingly, mechanobiology bore different mechanisms of mechanical modulation, ranging from highly localized stimuli, such as membrane indentation^7,8^ to inherently multi-cell stimulations, i.e., via acoustic waves^3,9,10,12^. Mechanical modulation is advantageous over electrical or optogenetic stimulation since it does not require an operational procedure for in vivo purposes nor alters the neuron’s genetic make-up^13^. Recent advances, such as focused ultrasound, made a great effort to mechanically modulate neuronal networks with high fidelity at mm spatial resolution^14^.

Although the initiation mechanisms have been extensively characterized^1,3,7^, it remains to be elucidated if mechanically affected neurons transmit signals to the neighbors. Tackling this question is particularly important for developing high-spatial resolution stimulation strategies, as the propagation of mechanical responses can adversely excite nearby non-target regions while concurrently promoting signal convolution in target areas. While the single-cell mechanostimulation focuses exclusively on the target cell’s responses^8^, the multi-cell modality impedes the direct estimation of signal interference^3^. There is a consensus that neurons respond internally to mechanical stimuli, as synaptic block did not alter the responses in multi-cell ultrasound stimulations^3,10^. This does not rule out cross-talk, as neurons communicate via alternative means, already found to disperse mechanoresponses between non-neuronal cells^15–17^. For example, paracrine signals^18^ and gap junctions^19,20^ are suited to exchange secondary messengers, which can regeneratively re-initiate responses in the neighbors^21^. Moreover, mechanical stimulation not only excites the neurons but also modulates the neurons’ excitability^12^. It is, therefore, crucial to evaluate whether mechanical stimulation exerts a neuromodulatory effect on non-target neurons, in a system that allows network screening during a single-neuron mechanostimulation. Reports indicate that single-neuron membrane deformation during the membrane-glass pipette interaction in patch-clamp induces calcium responses in target somatotropic pituitary neurons^22^. It remains to be elucidated if the effect is universal to the cortical neurons or if individual neuron patching exerts any broader effect on the neuronal network.

To test if target neurons disperse the mechanoresponses into the network, we investigated the effect of a pipette-mediated single-neuron deformation on a local in vitro network of cortical neurons. As a stimulus, we chose the membrane deformation during the giga-seal formation in patch-clamp while concurrently monitoring the surrounding network via calcium imaging. We demonstrate that the acute membrane indentation induces calcium plateaus in the target soma that persist in neurites and regeneratively propagate up to a few hundred microns in the network, to about a third of morphologically and activity-coupled neighbors. Calcium plateaus develop through calcium influx via MS and L-type voltage-gated calcium channels (Ca_v_s), independently of action potentials (APs). Our ground truth measurements show membrane depolarization in the target and responding neighbors, capable of eliciting APs. We show a non-synaptic, gap junction-mediated propagation with an average speed of 50 µm/s. Finally, we show that single-neuron mechanical perturbation imposes transient neuromodulatory effects on the manipulated and responding neurons. Our findings indicate that the spatial resolution of mechanical stimulation might be inherently limited and emphasize the temporal limitations for native network activity measurements post-giga-seal.

## Results

### Calcium plateaus arise during acute mechanical perturbation

**Figure 1:**
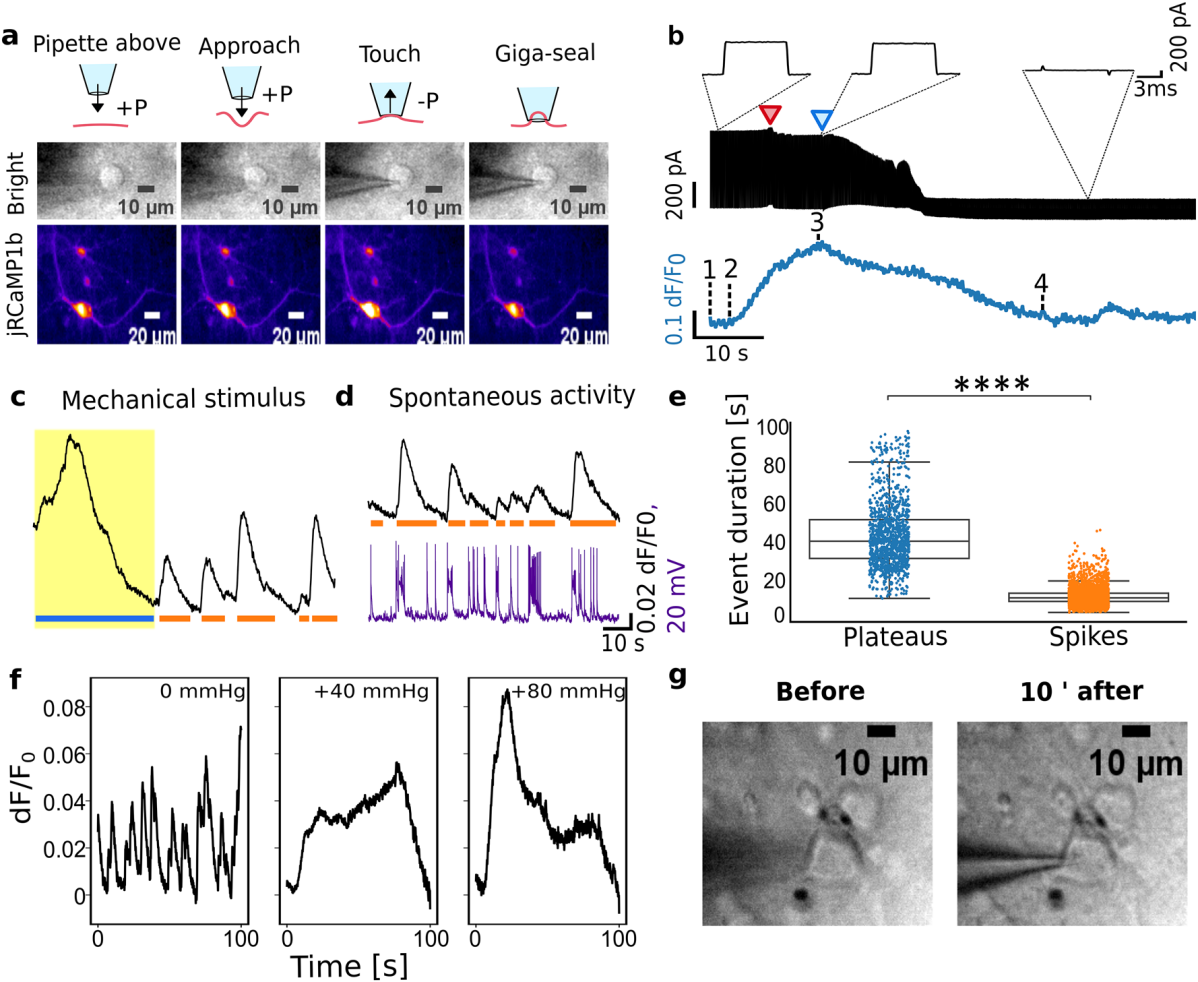
Calcium responses during membrane deformation. (**a**) Calcium plateaus coincide with the membrane indentation. Top: schematics of different steps during the giga-seal formation, middle: corresponding brightfield micrographs, bottom: jRCaMP signal. (**b**) Top: current responses to 5 mV, 5 ms, voltage pulses monitor the relative pipette position. The red arrow marks the initial membrane engagement with the pipette, followed by a steady increase in resistance due to further indentation; the blue arrow depicts the pressure release and suction, followed by giga-seal formation. Bottom: Corresponding calcium trace with numbers indicating phases in (**A**, **C**) Target trace during the membrane perturbation (yellow). (**D**) Calcium (black) and corresponding patch trace (purple) depicting the spontaneous activity of the neuron after the mechanical deformation in (**C**) Horizontal lines depict the duration of calcium spikes (orange) and plateaus (blue). (**e**) Duration of plateaus (N = 1291, blue) versus spikes (N = 4345, orange). Box plots span from the 1st to the 3rd quartile, and whiskers span over the 1.5 × IQR. Significance was estimated from two-tailed t-test (****:*p* < 10^–4^). (**f**) Calcium responses from the same neuron approached under no pressure, + 40 and + 80 mmHg overpressure. (**d**) Trypan blue staining pre- and 10 min post-giga-seal formation.

Calcium imaging via jRCaMP1b throughout the giga-seal formation revealed that the targeted neuron responds with slow calcium events. The responsiveness was robust, with every trial reliably evoking calcium increase in the target (Fig. 1a,b). Compared to AP-associated calcium spikes, these responses displayed slower, almost 5 × wider, kinetics (Fig. 1c–e) (41.71 ± 0.49 s, N = 1291 plateaus vs. 9.23 ± 0.07 s, N = 4345 spikes). We tested if calcium plateaus were evoked by diffusion of pipette solution (PS) or acute membrane deformation. Plateaus were present in control experiments with bath solution (BS)-containing pipette but were absent when PS was allowed to diffuse out of the pipette ca. 5 µm above the cell (Fig. S1). These demonstrate that acute mechanical perturbation, rather than diffusion of PS constituents, triggers the calcium plateau.

During the giga-seal establishment, the pipette approaches the neuron under positive pressure (∆P =  + 60–80 mmHg) and indents the membrane (Fig. 1a), followed by a pressure release and steady weak suction to promote the membrane protruding the tip. We evaluated whether indentation or protrusion triggered calcium plateaus. Paired electrical test-pulse recordings and calcium imaging during the membrane deformation showed that calcium plateau onset preceded the pipette-membrane engagement by 4.4 ± 1.3 s (N = 13), when the pipette was approximately 1 µm above the neuron (Fig. 1b). Calcium responses increased with further pipette advancement, reaching the maximum at the point of touch. Plateaus started declining after the pressure release (Fig. 1b, blue arrow) and returned to the baseline before the giga-seal.

Since Ca^2+^ onsets preceded the physical contact, we postulated that shear stress from the liquid triggers calcium plateaus. Under the no-pressure approach, weak calcium plateaus were masked by the neuron’s spontaneous activity, but overpressure led to the development of calcium plateaus (Fig. 1f). Plateau amplitudes correlated with the positive pressure applied, suggesting that neurons sense the magnitude of the mechanical stimuli. For + 60–80 mmHg overpressure, canonically used in in vitro patch-clamp, the calculated shear force was about 230 pN/µm^2^ (Supplementary). This lower boundary of applied mechanical forces is about 10–40 × stronger than localized membrane manipulations^7^. We excluded recordings showing signs of pipette-induced membrane damage to prevent interferences caused by local ion leakage around the penetration site (Supplementary Fig. S2)^23^. Experiments in trypan blue confirmed that the membrane didn’t rupture during our process (Fig. 1g), suggesting that calcium increases aren’t due to membrane injury.

**Figure 2:**
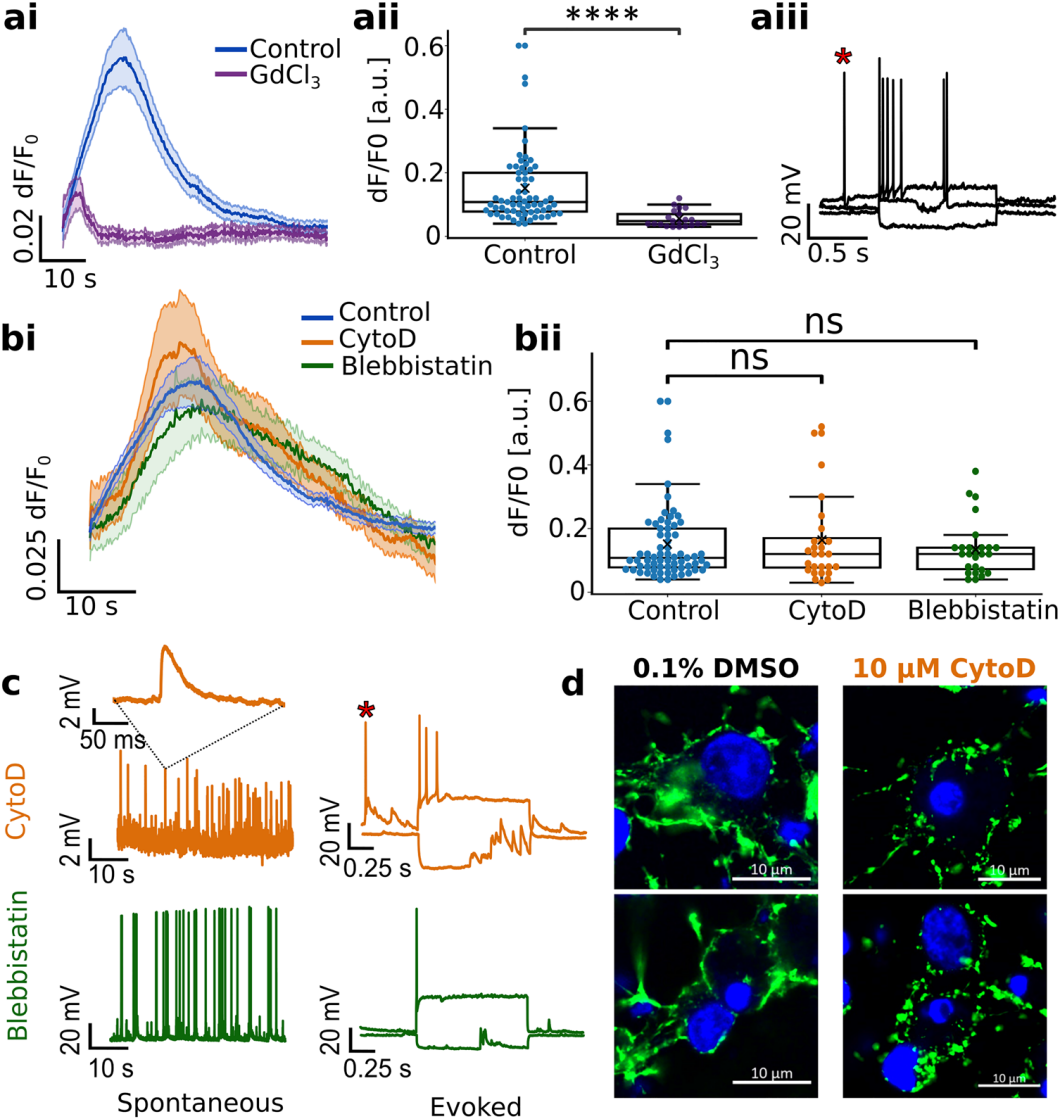
The role of the membrane and cytoskeleton in calcium plateau generation. (**a**) Universal block of MS channels via 50 µM GdCl_3_. (**ai**) averaged calcium plateaus from patched neurons in control (blue) and GdCl_3_-treated neurons (purple). (**aii**) calcium plateau amplitudes in control and treated group. (**aiii**) GdCl_3_ didn’t interfere with spontaneous (*-ed) or evoked firing. (**b**) Calcium responses to mechanical stimulation upon cytoskeleton disruption. (**bi**) averaged calcium plateaus of patched neurons in control (blue), cytoD (orange), and blebbistatin (green). Shaded areas represent standard errors. (**bii**) Patched neuron plateau amplitudes in control, cytoD, and blebbistatin treatments. (**c**) Pharmacological disruption of the cytoskeleton affected neither neuronal excitability nor synaptic communication. Intracellular recordings of neurons in cytoD and blebbistatin show intact synaptic communication and excitability; spontaneous APs preceding the stimulus, and post-synaptic potentials are also present (*-ed). (**d**) CytoD treatment disrupted the actin cytoskeleton. Green: phalloidin actin staining of 0.1% DMSO control (left) and CytoD group (right); blue: counterstaining of nuclei with DAPI. Box plots in (**aii**) and (**bii**) have boxes from the 1st to the 3rd quartile, the median as a horizontal line, the mean as an x, and whiskers spanning over a 1.5 × IQR. In both, significance was estimated via two-tailed Mann–Whitney test; *p*-value was adjusted for multiple comparisons using Bonferroni correction. ****: *p* ≤ 10^–4^ , ns: *p* > 0.5.

Gd^3+^, a broad-spectrum antagonist of MS channels, was used at 50 µM to prevent pleiotropic effects reported at higher concentrations^24^. A significant attenuation of plateaus in the Gd^3+^-treated group (0.055 ± 0.048; N = 20) confirms these responses initiate via MS channels. Moreover, spontaneous and current-evoked APs in Gd^3+^-treated neurons indicate that the attenuation is not due to Gd^3+^ interference with excitability (Fig. 2a).

Since actin cytoskeleton transfers the mechanical responses deeper into the cell^25^, we investigated if actin-mediated signaling cascades are involved in calcium plateau generation. 10 µM (0.1% DMSO) cytochalasin D (CytoD) prevented actin polymerization and disrupted the pre-existing cortex^26^. Unlike the 0.1% DMSO control, CytoD-treated neurons showed focal accumulations indicative of actin depolymerization (Fig. 2d). Previous work reported that CytoD may interfere with the cell’s membrane resistance and excitability^27^, however, it influenced neither excitability nor spontaneous activity (Fig. 2c). A treatment with 50 µM blebbistatin (–), a non-muscle MHC II ATPase inhibitor^28^, was applied to test if active actin reorganization is required. As with CytoD, blebbistatin failed to alter the neuron’s excitability and spontaneous firing (Fig. 2b). The amplitudes in treated groups were comparable to those of control (Fig. 2b): 0.12 (median, IQR: 0.1, N = 28) for CytoD, and 0.12 (median, IQR: 0.07, N = 26) for blebbistatin; control (median: 0.11, IQR: 0.124, N = 67). The persistence of mechanoresponses of cells with disrupted F-actin suggests that actin-mediated signal transduction is not a prerequisite for calcium plateaus. Instead, the signal initiates at the membrane, primarily through the activation of MS-channels.

### Regenerative propagation of calcium plateaus

**Figure 3:**
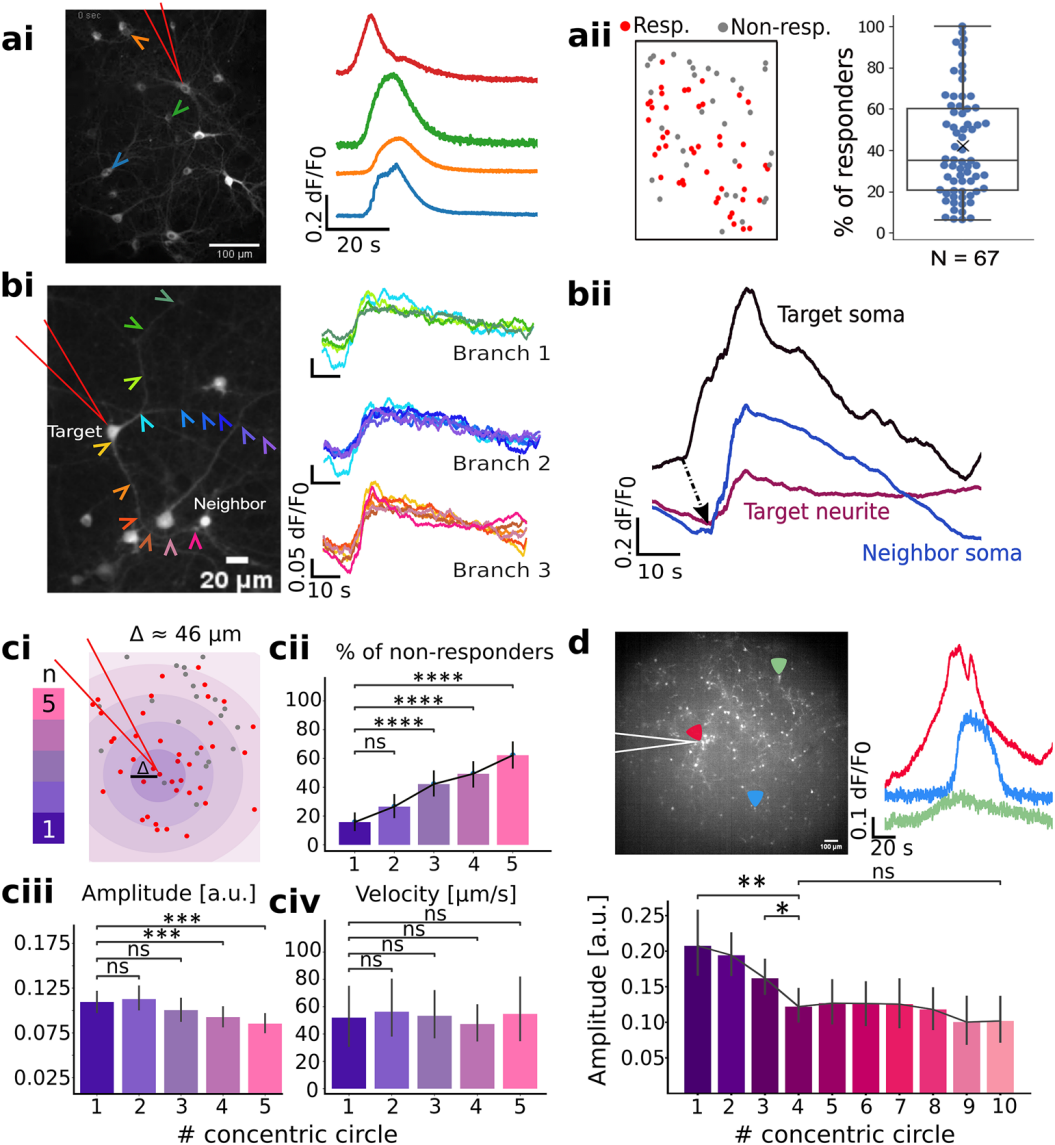
Regenerative propagation of calcium plateaus within the network. Long triangles point to the target in all plots. (**a**) Somatic calcium plateaus in 0.2 mm^2^ during the single-neuron deformation. (**ai**) Left: jRCaMP1b signal in 0.2 mm^2^, right: example ROIs marked with colored arrows. (**aii**) Left: a schematic overview of responding ROIs, right: fraction of responding neurons. Box plots have boxes from the first to the third quartile, the median as a horizontal line, the mean as an x, and whiskers spanning over a 1.5 × IQR. (**b**) Plateaus persist in neurites. (**bi**) Neurite calcium signals. Left: Target soma (1), neurite branches (arrows), and responding neighbors. Bottom scale: 30 µm. Right: Calcium plateaus from three neurite branches of the target. (**bii**) Plateau propagation from the soma through the neurites to the visibly connected neighbor. Bottom: somatic (black) and neuritic (purple) responses of the target and plateau from the neighbor’s soma (blue); onsets marked by a black arrow. (**c**) Calcium plateau propagation within concentric circles around the target. (**ci**) Schematic overview of responsiveness around the target (red). (**cii**) Non-responding fraction vs. distance. (**ciii**) Amplitude distribution around the target. (**civ**) Velocity of the waves crossing successive concentric circles. (**d)** jRCaMP1b signal in 3 mm^2^, with exemplary traces of the ROIs on the edges of the propagation. Bottom: Amplitude distribution around the target. Bar plots in (**c**) and (**d**) have errors spanning over 95% CI. Significance for pairwise comparisons was estimated via two-tailed Mann–Whitney or t-test, with p-values adjusted with Bonferroni correction; ns: *p* > 0.05, *: 0.01 < *p* ≤ 0.05, **: 10^–3^ < *p* ≤ 10^–2^, ***: 10^–4^ < *p* ≤ 10^–3^, ****: *p* ≤ 10^–4^.

Calcium imaging during membrane engagement was conducted in 67, 0.2 mm^2^ fields of view containing 51.8 ± 2.3 somas (3473 neurons). During the acute soma membrane deformation, about a third of the neighbors responded with calcium plateaus, similar in kinetics to the target (Fig. 3a). Despite the network inter-regional variability, the fraction of responders ranged from 6 to 100% (Fig. 3aii). We tested if this was a direct effect of shear stress from liquid leaving the tip. FITC added to PS showed that fluid exiting the tip is a sphere of only a few µm in radius (Supplementary Fig. S3). No FITC signal could be observed from the nearby responding assembly, suggesting the neighbors are not directly affected by the liquid flow (Supplementary Fig. S3).

In 18 out of 67 trials, the calcium signal from the target's neurites was well-isolated from the background. Responses initiated at the target soma persisted along the neurites and propagated to the neighbors (Fig. 3bi). Neurite signals had amplitudes 26.73 ± 3.02% of those from somas (N = 18), while the neighbors’ somatic signals were comparable to the target (Fig. 3bii). The attenuation may be due to reduced jRCaMP expression in the neurites^29^. A relatively low temporal resolution and reduced signal-to-noise ratio (SNR) prevented an accurate estimation of the intra-neurite signal propagation. However, the somatic signals consistently preceded the neurite signals. We estimated the intra-neuron propagation speed of 44.29 ± 9.08 µm/s (N = 18) from ∆t of somatic calcium plateaus and the farthest point in neurites. Likewise, in neighbors with distinct neurite signals, calcium plateaus propagated through the neighbor’s neurites and to the soma (Supplementary Fig. S4), with similar velocity: 46.15 ± 9.98 µm/s (N = 18). The findings suggest that responses evoked during the mechanical stimulation of individual neurons persist and propagate along the neurites and that the propagation route follows morphological connections. The latter was confirmed by mechanical stimulation in morphologically separated but proximal networks that didn’t respond to a nearby neuron deformation (Supplementary Fig. S5).

The fraction of plateauing neighbors fell with the distance from the target (*p* < 0.001, N = 67, Kruskal–Wallis) (Fig. 3b). However, the trend did not follow a simple radial distribution as from diffusion of a soluble messenger. Even the closest area around the target had about 20% of the non-plateauing neurons (Fig. 3). Propagation was about three orders of magnitude slower than the AP-associated calcium events and comparable to that of the neurons’ slow calcium waves^30^, or mechanically induced calcium waves in astrocytes^17^. We extracted the overall spread trends from concentric fields around the target, of common multiplier radius ∆r ~ 46 µm. There was no significant difference in velocities across the successive concentric circles: 51.9 ± 11.5 µm/s (1st circle), 56.3 ± 10 µm/s (2nd), 53.3 ± 8.8 µm/s (3rd), 47.3 ± 6.4 µm/s (4th), 54.7 ± 12.1 µm/s (5th) (*p*: 0.17, N = 67, Kruskal–Wallis). Signal amplitude decreased farther from the target (*p* < 0.01, N = 67, ANOVA), starting from ∆F/F0 = 0.109 within the first circle to ∆F/F0 = 0.085 in the fifth. Despite the slight decline, amplitudes in the last two regions remained relatively constant.

Plateauing neighbors were observed on the edges of the 0.2 mm^2^ field of view, so we investigated the propagation limit in a ca. 3 mm^2^ area. These were exclusively to estimate the extent of propagation due to technical limitations of the temporal resolution. Plateau propagation had an average end-boundary of about 730 µm (Supplementary Fig. S6). Accordingly, the amplitude significantly declined at the fourth circle, but remained consistent thereafter. The last responding circle contained neurons that either responded with a developed calcium plateau or remained silent. (Fig. 3d). The absence of exponential amplitude decay with distance and preserved velocity strongly suggest that calcium plateaus propagate in a regenerative fashion.

### Ground truth and plateau initiation mechanism

**Figure 4:**
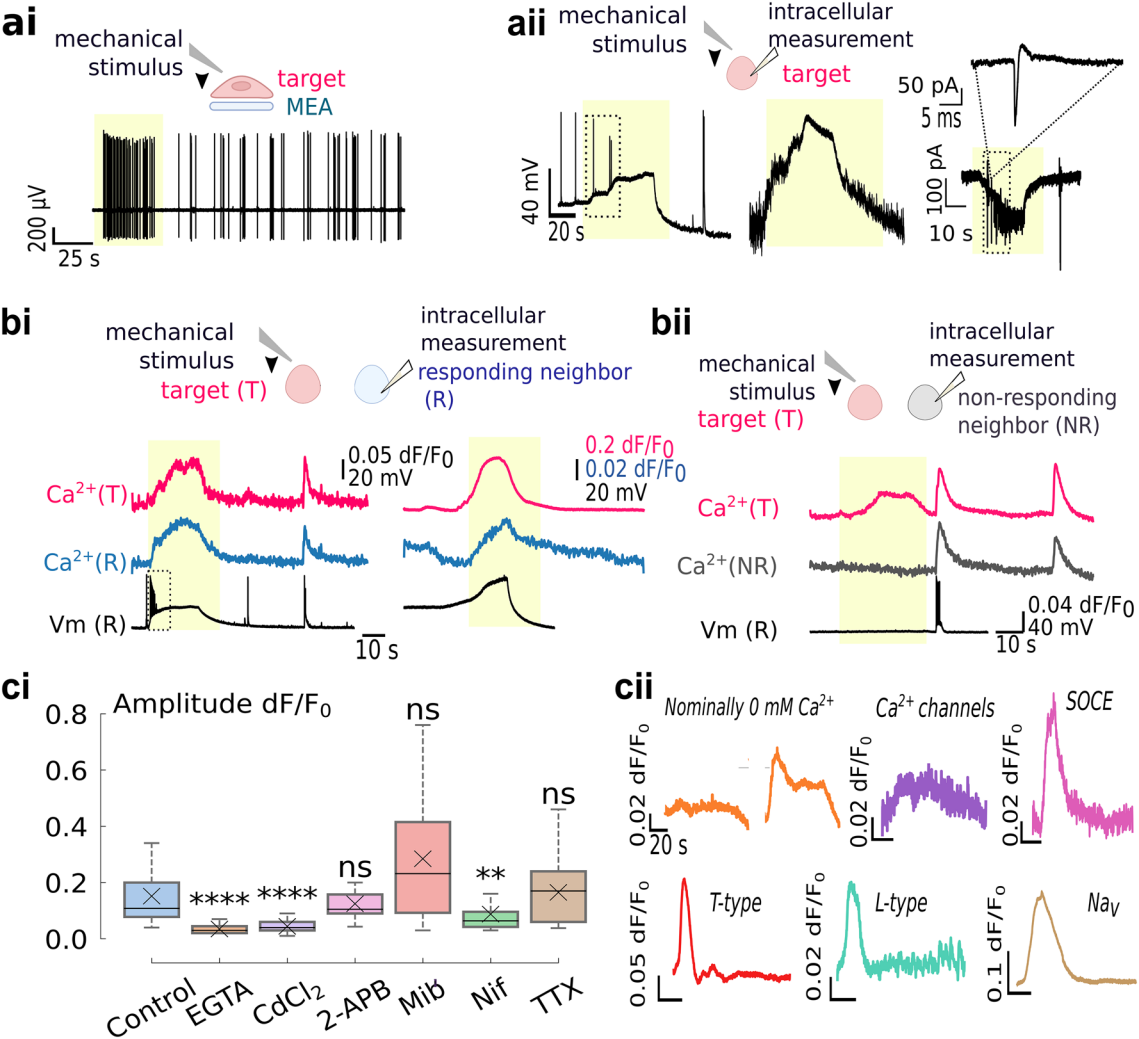
Calcium plateau generation and ground-truth. (**a**) Ground-truth of mechanical responses at the target. Yellow rectangles emphasize the mechanical stimulus response. (**ai**) On-chip patch-clamp measurements show bursting activity on the electrode coupled to the deformed neuron. (**aii**) Intracellular voltage and current responses to mechanical deformation showing slow depolarizations and inward currents, capable of inducing APs. (**b**) Ground-truth of calcium plateaus during the deformation of a nearby cell. (**bi**) Responding neighbors (R) displayed depolarizations corresponding to calcium plateaus, capable of eliciting APs (left, rectangle). (**bii**) Non-responding neurons lacked the calcium responses and respective depolarizations and showed only AP-associated calcium activity. (**c**) Pharmacological investigations of the calcium plateau generation. (**ci**) Plateau amplitudes at the target in control and treated groups. (**cii**) Exemplary target traces under pharmacological treatments, colors match (**ci**) For EGTA in 0 mM Ca^2+^ (orange), calcium traces with non-significant SNR (left) and fully-developed plateaus (right) are shown. Box plots have whiskers spanning over 1.5 × IQR. Significance was tested via two-tailed Mann–Whitney or t-test, with p-values adjusted with Bonferroni correction; ns: *p* > 0.05, **: 10^–3^ < *p* ≤ 10^–2^, ****: *p* ≤ 10^–4^.

Extracellular electrophysiology tested the network AP activity during acute single cell deformation. Microelectrode array (MEA) measurements showed coincident mechanical stimulation and bursting activity (Fig. 4ai). Corresponding bursts occurred in the neighboring electrodes, similar to the end-boundary of the observable calcium plateau (Supplementary Fig. S7). To evaluate the target’s membrane potential (Vm) changes at a subthreshold resolution, we performed two consecutive contacts separated by 10 min: the first pipette detected subthreshold changes upon deformation via a second pipette. These indicated slow depolarization associated with the mechanical stimulus, which in 2 out of 5 recordings led to firing activity (Fig. 4aii). In voltage-clamp mode, slow inward currents were superimposed with the AP-associated fast currents. These confirmed the mechanostimulation’s capability to directly evoke APs.

Random pairs of neurons were chosen to detect Vm changes during the mechanical deformation of the neighbor. Non-plateauing neighbors were either silent during the mechanical stimulus of the target, or showed only AP-associated calcium spikes uncorrelated with the stimulus (Fig. 4bii). In contrast, plateauing neurons had slow depolarizations similar to the target neuron’s response (Fig. 4bi). These depolarizations matched the kinetics of calcium plateaus and induced APs in 45% of the responders (Fig. 4bi). The firing probability correlated with the initiation kinetics and magnitude of depolarization. Specifically, the up kinetics of the bursting neurons were 12 × faster (median: 61.74 mV/s, IQR: 53.17 mV/s, N = 10) than non-bursting neurons (median: 4.92 mV/s, IQR: 4.19 mV/s, N = 12, *p*: 8.73 × 10^–5^, two-tailed Mann–Whitney, Fig. S8).

Na_v_s do not participate in plateau generation, as treatment with 1 µM TTX^31^ failed to attenuate calcium responses (dF/F_0_ = 0.16 ± 0.02, N = 21; *p*: 1.00, two-tailed Mann–Whitney Fig. 4c). TTX inhibited APs but not calcium plateaus (Supplementary Fig. S11), confirming that calcium plateaus don’t require APs for a generation. Rather, mechanical stimulus triggers depolarizations that may elicit APs. The consistency of the target’s response ruled out the eventual between-trial differences as a cause of different depolarization kinetics, and the neuron's distance from the initiation site didn’t indicate whether the plateauing neighbor fired APs (Supplementary Fig. S9). These validate that depolarization kinetics and firing probability are due to intrinsic neuronal variability. Further analysis of passive membrane properties showed no significant dissimilarities between the bursting and non-bursting neurons (Supplementary Fig. S9), suggesting that subtle differences in molecular constitution might dictate depolarization kinetics and the firing outcomes.

MS channels also interact with Ca^2+^ leak channels from the endoplasmic reticulum^25,32^, and we evaluated if plateaus originated via calcium influx or mobilization from internal stores. Neurons were measured in a bath with 2 mM EGTA for nominally 0 mM Ca^2+^. Mechanostimulation evoked calcium plateaus in only 30% of treated neurons, and the rest had a non-significant SNR. Those that plateaued had about 80% amplitude decrease compared to the control (dF/F_0_ = 0.035 ± 0.003, N = 15, *p*: 1.25 × 10^–7^, two-tailed Mann–Whitney), suggesting the plateaus develop mainly through external Ca^2+^ influx. Incomplete abolition is unsurprising, as previous work reported incomplete chelation at 2 mM EGTA^33^. Neurons were treated with Cd^2+^ to confirm the extracellular origin of calcium. Cd^2+^ blocks 60% of calcium currents at a resting Vm and fully blocks at + 20 mV^34^. In 200 µM Cd^2+^, plateau amplitude reduced to 70% of the control group (dF/F_0_ = 0.04 ± 0.02, N = 19; *p*: 8.24 × 10^–8^, Mann–Whitney). The weak signals could be due to an incomplete block at the resting Vm advancing further as the weakened calcium plateau develops.

As store-operated calcium entry (SOCE) is also found to mediate calcium influx in neurons, mechanical perturbation was repeated in 2-APB. As plateau amplitudes preserved in 100 µM 2-APB (median dF/F_0_: 0.105, IQR: 0.07, N = 26; *p*: 0.85, Mann–Whitney), Ca^2+^ influx is likely unrelated to SOCE. Application of 10 µM Nifedipine, an L-type calcium channel antagonist^35^, reduced the plateau amplitudes by almost 50% (dF/F_0_ = 0.087 ± 0.016, N = 18; *p*: 0.002, two-tailed Mann–Whitney). There was no statistically significant difference in amplitudes of the mibefradil treatment that in 5 µM preferably targeted T-type over L-type Ca_v_s (dF/F_0_ = 0.284 ± 0.059, N = 14; *p*: 0.29, two-tailed Mann–Whitney), indicating that T-type voltage-gated calcium channels aren’t required in signal initiation^36^. These suggest the neuron’s mechanoresponses initiate via an external calcium influx through MS and L-type Ca_v_ and induce depolarization capable of eliciting APs.

### Gap junctions propagate mechanical responses within the neuronal ensemble

**Figure 5:**
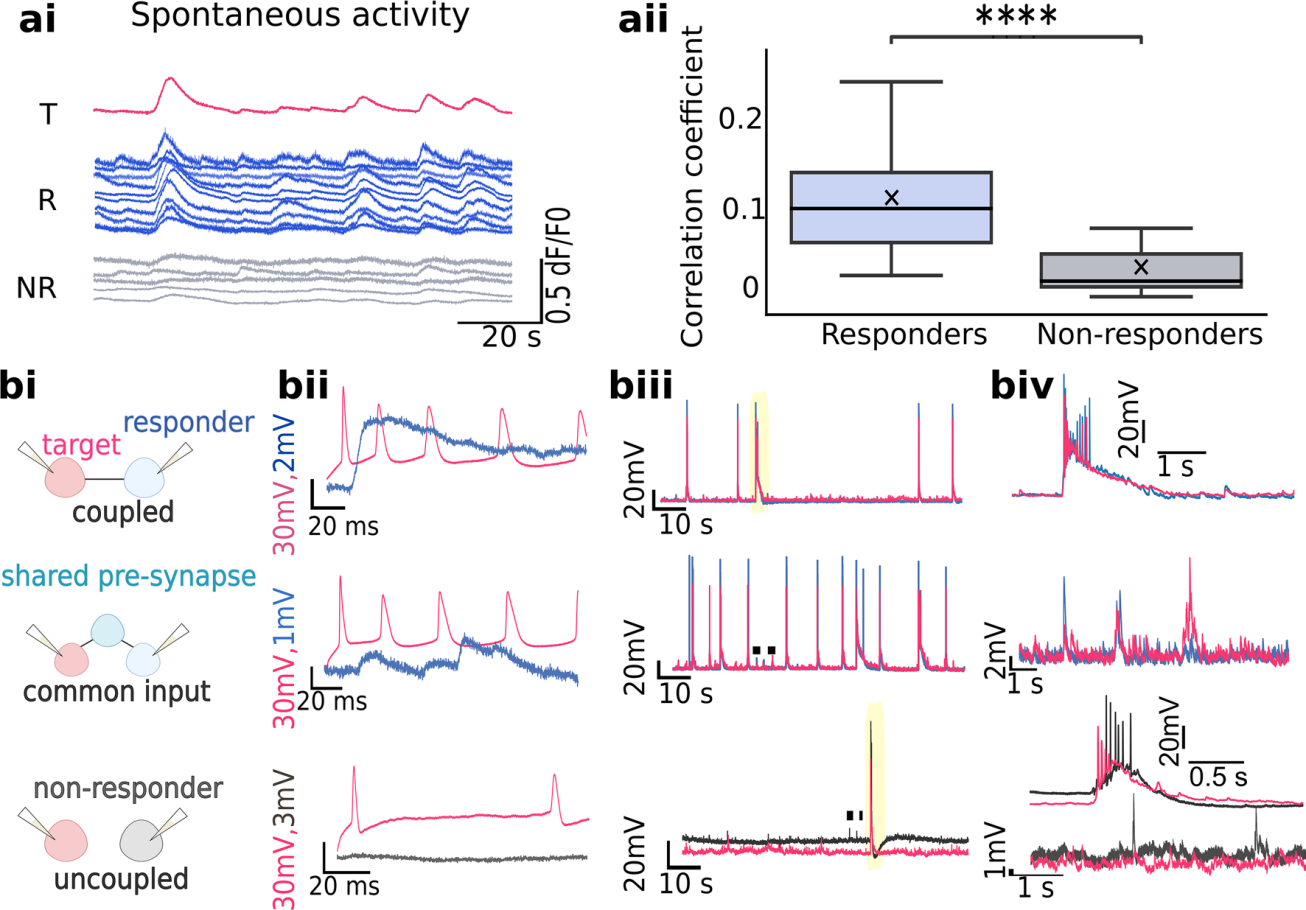
Mechanical responses propagate through a defined neuronal ensemble. (**a**) Functional connections from calcium imaging of spontaneous activity prior to mechanical stimulation. (**ai**) Exemplary calcium traces of the target (T), responders (R), and non-responders (NR) before the target deformation. R/NR labelled after mechanical stimulus measurement. (**aii**) Box plot showing cross-correlation coefficients of spontaneous pre-stimulus calcium spikes, with whiskers spanning over 1.5 × IQR. ****: *p* ≤ 10^–4^, estimated via two-tailed Mann–Whitney test. (**b**) Direct connectivity estimations through paired whole-cell measurements following the deformation of the target. (**bi**) Schematic overview of different configurations; neurons responding to a neighbor’s deformation (top and middle) are in blue, non-responders are in gray. (**bii**) Neighbor’s responses to APs evoked via electrical stimulation. Rows match schematics depicted in (**bi**). (**biii**) Spontaneous activity detected intracellularly from two patched neurons. Biv. Focus on bursting events (yellow in (**biii**)) or individual post-synaptic potentials (dotted in (**biii**)).

To assess if the responding assembly represents a functional community, we estimated how its spontaneous calcium activity prior to mechanical stimulus relates to the target. As shown in Fig. 5, averaged correlation coefficient within the responding assembly (median: 0.1, IQR: 0.08, N = 24) was about 10 × higher than between the target and non-plateauing neurons (median: 0.01, IQR: 0.03, N = 24; *p*: 3.33 × 10^–7^, two-tailed Mann–Whitney). The extent of activity overlap between the target and non-responders was high during the network-wide bursts, where about a half of the non-responders also contributed (Supplementary Fig. S10). Because also uncoupled neurons correlate during the network bursts (Fig. 5bii), connectivity was directly estimated via paired whole-cell measurements following the mechanical stimulation. The pooled fraction of coupled neurons (53%, N = 19) fits in the range of connectivity reported for neuronal networks^37^. Of 13 investigated pairs belonging to the responding assembly, 10 were bi- or unidirectionally coupled, and the rest only shared a pre-synaptic neighbor (Fig. 5bi). Among the non-responders, no neurons coupled to the target, and none shared spontaneous events outside of bursts (N = 6).

**Figure 6:**
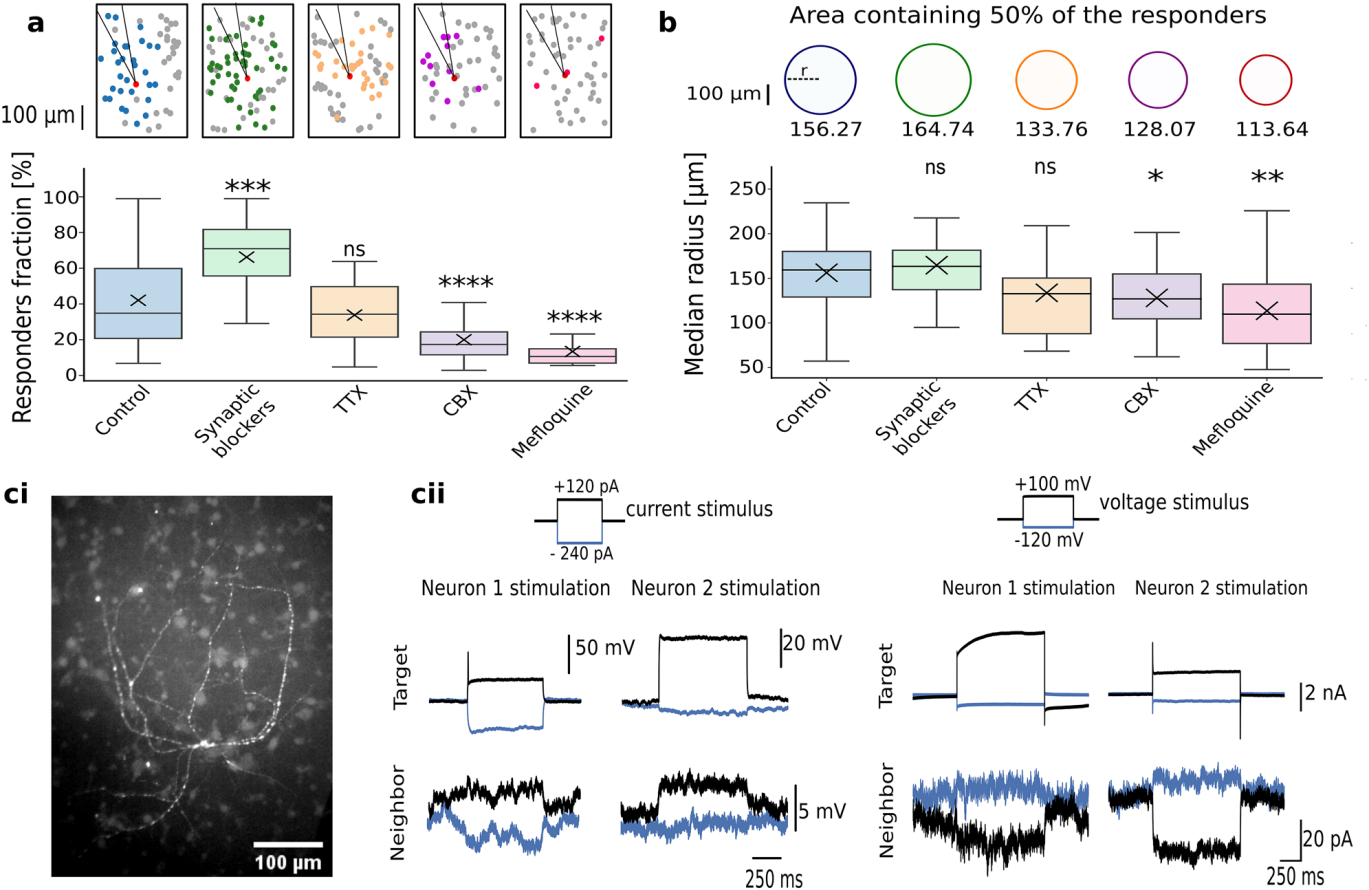
Gap junctions as the main route for calcium plateau propagation. (**a**) Pharmacological treatments to investigate mediators of propagation. Top: representative schematics of 0.2 mm^2^ network depicting relative distributions of responding (color) and non-responding neighbor (gray) upon a target neuron (red) deformation. Color-code matches the box plot below. Fraction of responding neurons in 0.2 mm^2^ in untreated group, synaptic blocker cocktail, TTX, CBX and mefloquine. (**b**) Estimating the propagation extent upon different pharmacological treatments. Top: Areas around the target containing 50% of the responding assembly, colored to match the box plot. Numbers depict the average circle radii in µm. (**c**) Confirmation of gap junctions in in vitro networks of cortical neurons. (**ci**) Neurobiotin injection visualized by streptavidin-Alexa 488 conjugate. (**cii**) Paired intracellular recordings show signal transmission through electrical junctions upon a current (left) and voltage injection (right). Box plots in a and b have whiskers spanning over the 1.5xIQR. Symbols above the whiskers denote p-values from pairwise comparison via two-sided Mann–Whitney, adjusted with Bonferroni correction; ns: *p* > 0.05, *: 0.01 < *p* ≤ 0.05, **: 10^–3^ < *p* ≤ 10^–2^, ***: 10^–4^ < *p* ≤ 10^–3^, ****: *p* ≤ 10^–4^.

Synapse involvement was tested in an antagonist cocktail containing (µM): NBQX (20), D-AP5 (80) and bicuculine (20)^38^. These didn’t attenuate calcium plateaus (Supplementary Fig. S12) nor reduce the number of responding neurons (Fig. 6a). In contrast, synaptic blockers increased the fraction of responding neurons (66.77 ± 4.18%, N = 26, *p*: 2.09 × 10^–4^, two-tailed Mann–Whitney). A higher fraction of responders was not due to removal of inhibition, since treatment with bicuculine alone failed to increase the fraction of responding neurons (median: 29.17%, IQR: 30.41%, N = 15, *p*: 1.00, two-tailed Mann–Whitney). These suggest that the synaptic influx and mechanical responses are temporally exclusive. We postulate that the coincident synaptic influx interferes with calcium plateau regeneration, either by shunting inhibition or homeostatic mechanisms aiming to reduce over-excitation. Application of TTX to block APs also did not reduce the fraction of responding neurons (33.83 ± 3.99%, N = 21, *p*: 0.98, two-tailed Mann–Whitney).These confirm that calcium plateaus propagate independently of APs and chemical synapses.

As recent findings point to the ubiquitous presence of gap junctions^20^, we tested if they mediate the inter-neuron propagation. We confirm the gap junctions in our cultures through tracer injection and paired electrophysiology. Neurobiotin diffusion into the neighbors (Fig. 6c) demonstrated physical coupling. The intracellular measurements of close neuronal pairs in synaptic blockers confirmed passive electrical responses were detected only in spatially-distinguished pairs of somas (Fig. 6c). Upon a voltage injection, the neighbor’s currents were of opposite sign, reflecting the neighbor’s charging when the stimulated neuron discharged, and vice versa. Additionally, consecutive mechanical deformations of neuron pairs show cross-responsiveness and bi-directional propagation of mechanoresponses, pointing to reciprocity typical for gap junctions (Supplementary Fig. S13).

Without affecting the amplitude (Fig. S12), connexin antagonist carbenoxolone (CBX) halved the responding neighbors (median: 17.14%, IQR: 12.99, N = 29, *p*: 8.4 × 10^–5^, two-tailed Mann–Whitney), while the more potent antagonist mefloquine^39^ reduced the fraction of responding neurons by 70% (median: 10.28%, IQR: 8.13, N = 22, *p*: 2.2 × 10^–7^, two-tailed Mann–Whitney) of the control. It is worth mentioning that mefloquine also antagonizes the L-type Ca_v_s. However, the intact amplitudes suggest that the reduced network responsiveness was not due to its effects on response regeneration. Among investigated treatments, only blocking gap junctions limited the extent of the responding assembly (Fig. 6b). The findings point to the gap junctions as main mediators of calcium plateau propagation, and the current model proposes the exchange of the secondary messengers, which then re-initiate the plateau in the recipient.

### Single-neuron deformation transiently modulates the responding ensemble

**Figure 7:**
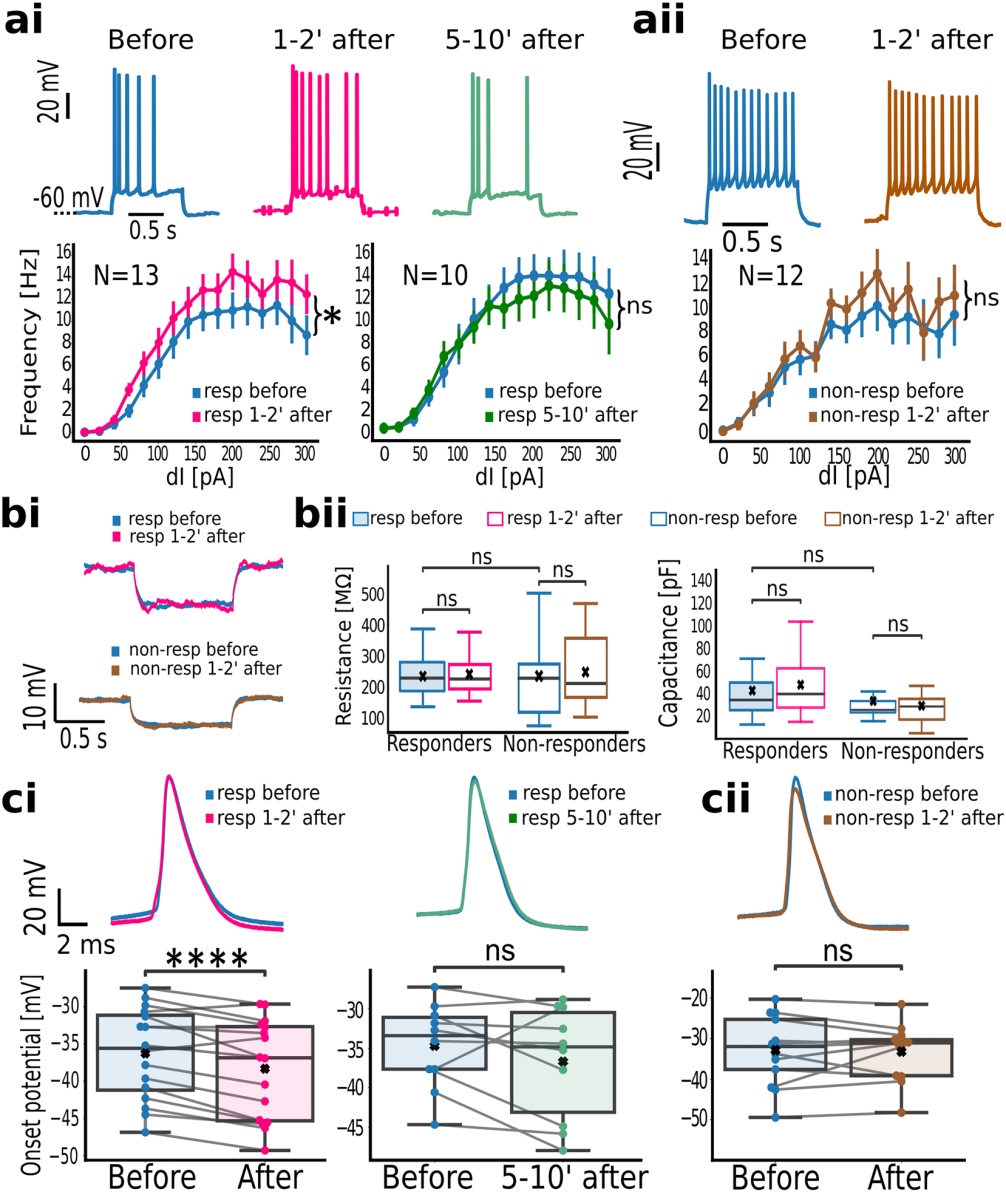
Estimating intrinsic excitability in neurons that responded to the neighbor’s mechanical deformation. (**a**) Intrinsic excitability changes throughout the mechanical stimulation of a nearby neuron. (**ai**) Evoked firing of responding neuron before (blue), 1–2 min after (pink) and 5–10’ after (green) the neighbor deformation. Bottom: firing frequency per applied current 1–2 min, and 5–10 min after the mechanical stimulus. (**aii**) Evoked firing of the non-responding neurons before (blue) and 1–2 min after the mechanical stimulus (brown). Bottom: firing frequency comparison before and 1–2 min after the stimulus. Whiskers denote standard errors. (**b**) Passive membrane properties among responding and non-responding neurons during a nearby neuron stimulation. (**bi**) Exemplary voltage responses to a − 20 pA pulse detected, for plateauing and non-plateauing neurons. (**bii**) Membrane resistance (left) and capacitance (right) for responding and non-responding neurons, before and after the mechanical stimulation. (**c**) Comparison of active neuron properties before and after the mechanical stimulus. (**ci**) Action potential waveforms of plateauing neurons before (blue), 1–2 min after (pink) and 5–10 min after the mechanical stimulus (green) and firing thresholds. (**cii**) Action potential waveforms among non-plateauing neurons before (blue) and after (brown) the mechanical stimulus and threshold comparison on the bottom. Whiskers of the box plots span over 1.5 × IQR. Dependent samples were probed for significance via Wilcoxon or paired t-test; independent samples were tested via two-tailed, two-sample t-test: ns: *p* > 0.05, *: 0.01 < *p* ≤ 0.05, ****: *p* ≤ 10^–4^.

We investigated if prolonged calcium increases during the single-neuron deformation had a neuromodulatory effect on the responding ensemble. The intrinsic excitability was tested from membrane responses to the current injection. As a baseline, the excitability was probed 5 min before the deformation of a randomly chosen neighbor and compared to responses after. Based on their responses to a neighbor’s deformation, the probed neurons were divided into plateauing or non-plateauing. Deforming a nearby neuron increased the firing frequency of the plateauing multispiking neighbors from 8 ± 1 to 10 ± 1 Hz (N = 13, *p*: 3.212 × 10^–4^, paired t-test, Fig. 7a). The firing frequency returned to the baseline 10 min after the neighbor’s deformation (9 ± 1 vs. 9 ± 1 Hz; N = 10, *p*: 0.406 paired t-test). In contrast, the firing frequency of the non-responders wasn’t affected by deformation of a nearby neuron (7 ± 1 vs. 8 ± 1, N = 12, *p*: 0.445, paired t-test). A similar firing frequency of the responding and non-responding multispikers (*p*: 0.489, two-sample t-test) suggests that the observed frequency changes aren’t pre-defined by the firing type. The absence of the effect among the non-responders indicates a response-specific potentiation of pre-synapses for further inputs.

The voltage responses to a − 20 pA current pulse were used to estimate membrane resistance and capacitance. First, we show that responding and non-responding neurons didn’t differ in passive membrane properties (Fig. 7b). Mechanical stimulation of a nearby neuron did not alter membrane resistance in the responding (236 ± 22–243 ± 17 MΩ, N = 16, *p*: 0.732 paired t-test) nor non-responding group (237 ± 37–250 ± 33 MΩ, N = 13, *p*: 0.617 paired t-test). Similarly, the membrane capacitance remained fairly constant in both groups after the stimulation (responders: 43 ± 7–30 ± 5, *p*: 0.74, Wilcoxon; non-responders: 34 ± 5–30 ± 4 pF, *p*: 0.364, Wilcoxon). These indicate the intrinsic excitability changes aren’t due to modulation of the constitutive membrane conductivities, or effective membrane area.

In plateauing neighbors, mechanical stimulation reduced the firing threshold (− 36.27 ± 1.53 to − 38.33 ± 1.66 mV; N = 16, *p*: 5.535 × 10^–4^, paired t-test; Fig. 7c), while the firing of non-plateauing neurons was unaffected (− 32.86 ± 2.37 to − 33.20 ± 1.91 mV, N = 13, *p*: 0.784, paired t-test). This selective modulation of the neuron’s onset was transient, as there was not a significant difference 10 min after the mechanostimulation of the neighbor (− 36.68 ± 2.27 to − 34.69 ± 1.70 mV, N = 10, *p*: 0.206, paired t-test). In addition, the firing threshold was similar between the firing and non-firing group (*p*: 0.22, two-sample t-test). These suggest that the increased gain of the responding ensemble post-stimulation is likely due to the modulation of sodium channel opening probability by the internal calcium increase.

## Discussion

We demonstrate that acute single-neuron deformation during the giga-seal evokes the calcium responses that regeneratively propagate, excite, and modulate the target and coupled neighbors. The findings challenge the established model of the neurons’ individual responses to mechanical stimuli, with relevant implications for developing mechanical neuromodulation strategies. The current model implies that spatial resolution is determined solely by the stimulus region. Accordingly, the recent advances were mainly technical, through stimulus optimization to promote the spatial specificity^14^. Our in vitro single-neuron focused study demonstrated response dispersion up to a few hundred microns, which influences the design of future experiments and therapeutic approaches aiming to operate at a submillimeter resolution. To our best knowledge, this is the first report of mechanical responses propagating through the network of neurons. The reduced network responsiveness and propagation extent in gap junction blockers suggests the gap junctions are a target to sequestrate the responses and reduce the cross-talk within the target regions. The calcium influx is via MS channels at the membrane, as plateaus did not require ER engagement nor cortical actin-mediated force transmission to the deeper structures, such as substrate-interfacing integrins. Plateaus further develop via L-type but not T-type Ca_v_s, independently of APs.

As shown by recent studies of ultrasound-evoked calcium responses, several MS species jointly and even synergistically contribute to calcium influxes^3^. Besides the mechanically gated ion channels (Piezo^40^, two-pore domain K_2_P family^41^), membrane deformation modulates the gating of several ion channel species, including voltage-^42^ and ligand-gated channels^43^, G-protein coupled receptors^44^, and multi-modal transient receptors^45^. This rapid mechanosensitivity allows prompt responses to acute mechanical cues during the neuritic outgrowth and network wiring^8,46^, provides a homeostatic platform for osmotic disturbances^47^, and offers prompt regulation of neuronal plasticity^48^. MS channels are also early responders to pathological membrane deformations, such as the traumatic brain injury (TBI)^49^. Our findings suggest that calcium influxes from localized impacts have a wider spread, further amplified by TBI-associated increases in gap junction conductivity^50,51^. Future studies should thus determine the magnitude of stimulation and number of cells affected for long term changes in gap junction expression and their alteration of the network’s processing of stimulation.

Mechanosensitive integrins in focal adhesions are found to mediate and modulate internal calcium influxes^52–54^, by activating several kinase pathways. However integrins in our stimulation were not potentiated, as we stimulate the free membrane in medium with non-functionalized glass. While we can not rule out the modulatory effect of integrins, the rapid plateau development contrasts previously demonstrated integrin-mediated calcium responses, which are delayed by tens to hundreds of seconds^52,54^.

To avoid the confounds from the pipette, the excitatory effect of mechanical stimulus is standardly addressed by imaging with limited amplitude and temporal resolution^3,7,9,10^, which may not fully capture the intricacies of the neuronal firing. This study harnessed the regenerative propagation of mechanical responses to assess the capacity of mechanostimulation in evoking APs in neurons that were not subjected to deformation but responded with calcium plateaus. This benchmarking revealed that sustained mechanically evoked calcium influxes produced APs only in half of the affected neurons, suggesting that the firing causality is only secondary. These should be taken into account particularly when mechanostimulation is intended to evoke APs.

The primary propagation mechanism may be via molecular pathways triggered and amplified by internal calcium increase. The slow mechanical responses disperse more due to the bi-directional, non-quantal and neurotransmitter-independent nature of gap-junctional propagation^55^. The propagation extent is likely similar in higher levels of neuron network organization, as functional gap junctions were also detected in adult brains, both in vivo and in vitro^20^. Although synapses were not required to propagate mechanical responses, we found that the local network’s responsiveness (but not response magnitude) is significantly greater when synapses were silenced. We attribute the exclusion of synaptically-driven network synchronicity and gap-junction mediated calcium plateaus to shunting inhibition at the recipient from synchronous synaptic inputs, which is abolished when synapses are blocked. This mutual exclusion, also found for spontaneous but co-existing propagation routes^38^, suggests the degree of mechanoresponse dispersion will depend not only on the morphological wiring, but also on the strengths of synaptic connections.

The extent and specificity of response propagation suggest that single-neuron deformation can be invaluable for accurate neuronal ensemble mapping. Firstly, the calcium responses to a single-neuron deformation are easily detected by calcium imaging, due to strong calcium influxes that propagate regeneratively. In our dense in vitro networks, electrically stimulated APs triggered PSPs without APs in the recipient, which is unsurprising since synaptic strength scales inversely with cell density. Since these PSPs are poorly visualized by calcium imaging, mapping based on electrical stimulation omits many true connections. Secondly, despite the lower action potential yield in the target compared to optogenetic or electrical stimulation^56^, the dispersion extent of mechanical stimulation is greater. In canonical transmission, the membrane’s length constant prevents the passive propagation of subthreshold events, and whether a postsynaptic cell fires depends on the neurotransmitter, synaptic strength, and summation of other inputs^57^. In contrast, mechanical response persists, propagates through the neuron without attenuation, and re-initiates in the neighbor, to visualize more connections.

Mechanically evoked calcium waves have a selective neuromodulatory effect on the responding ensemble. Only the defined neuronal subpopulation that responded to the deformation of the nearby neighbor showed a transient increase in gain and excitability. The current model proposes calcium influxes triggered by the single-neuron deformation affecting the AP generation in the target, as well as the coupled neurons. Such mechanisms may involve modulations of voltage-gated conductances, previously found to mediate intrinsic excitability of pre-synapses during the spike-timing related plasticity (STDP)^58^. Despite sharing a similar potentiation mechanism, opto-eletrically evoked ensemble potentiations require repetitive and synchronous co-activations of many coupled neurons to exert an effect; these requirements are likely from limited calcium influxes associated with AP-driven electrotonic changes^59^, and NMDARs acting as coincident detectors^60^. In contrast, mechanically evoked calcium influxes establish short-term ensemble potentiation, when effectively a single neuron is deformed. Since a single-time mechanical stimulation suffices to introduce transient excitability modulations, one future goal is to estimate whether a repetitive, but localized, mechanical stimulation can be used for functional network re-programming by targeted ensemble potentiation. It would also be of interest to evaluate the neurons recruitment by potentiating previously silent synapses.

Finally, the mechanical stimulus in this study was the membrane deformation associated with giga-seal in patch-clamp, previously shown to excite target pituitary neurons^22^. For the first time, our study demonstrates giga-seal excitation in cortical neurons, beyond the target, along with the transient neuromodulatory effect. The latter affects the native intrinsic excitability estimates and introduces confounds in network responses to electrical stimulations, especially when the activity is sampled inconsistently in close windows following the giga-seal formation. Through a detailed characterization of the dynamics and extent of calcium responses, we propose a wait time of 5–10 min post-giga-seal formation, after which activity and network measurements are reliable when patch-clamp is involved.

## Materials and methods

### Ethics statement

Primary neurons were isolated in accordance with the German Animal Protection Law (TSchG). All experimental protocols were approved by the local animal ethics committee (Landesumweltamt für Natur, Umwelt und Verbraucherschutz (LANUV), Nordrhein-Westfalen, Recklinghausen, Germany; #81-02.04.2018.A190). All methods were performed in accordance with relevant guidelines and regulations and are reported in accordance with ARRIVE guidelines.

### Neuronal culture

Cortical neurons were isolated from E18 Wistar rats (RjHan:WI, supplied by Janvier) of both sexes. Following a 10 min 0.05% trypsin treatment, dissociated neurons were plated at 750 cells/mm^2^ on fire-sterilized and PLL-coated coverslips. Incubation was at 37 °C and 5% CO_2_, in Neurobasal medium (NB) supplemented with 0.5 mM glutamine, 1% B27 and 100 µM gentamycin. Culturing conditions favored neurons over glial growth. Medium was half-exchanged twice per week and measurements started on DIV 21. For calcium imaging, neurons were transduced with on DIV 14 (MOI: 2 × 10^5^ GC/cell), followed by a full-medium change 3 days post-transduction. pAAV.Syn.NES-jRCaMP1b.WPRE.SV40 was a gift from Douglas Kim & GENIE Project, AAVs were produced by Addgene #100851^61^. The transduction efficiency was estimated to be 81%. Ethanol sterilized and PLL-coated MEAs recorded neuron activity from DIV 14. All measurements were conducted at room temperature (RT).

### Calcium imaging

All measurements were done on 21–29 DIV neurons, in bath solution (BS, containing (in mM): NaCl (120), KCl (3), MgCl_2_ (1), CaCl_2_ (2), HEPES (10), at pH = 7.3 adjusted with NaOH). Imaging was under an AxioScope upright microscope (Carl Zeiss AG) coupled to a Zyla sCMOS camera (Andor). For illumination, an HBO 100 W lamp (Zeiss), with 153.7 mW/cm^2^ power density was used. Detection was via BP 546 $\pm$ 12 nm excitation and LP 590 nm emission filter. A 20 × UMPlanFl objective (Olympus) was used in 0.2mm^2^, while a 10 × air objective Epiplan (Carl Zeiss) was used for 3 mm^2^ fields of view. Acquisition was at 50 Hz, with 0.02 s exposure and 4 × 4 binning.

Processing was through a custom-made analysis pipeline^62^. ROIs were automatically detected through maximum intensity projection-defined borders, that were manually curated in the SamuROI GUI^63^. Baseline (F_0_) was defined by the 5th percentile of a 2000 frame wide sliding window. Mean intensity traces were normalized to F_0_ and filtered by a 3 Hz Butterworth low-pass filter.

### Patch-clamp and calcium imaging measurements

Whole-cell patch-clamp was done in BS. Micropipettes were pulled on a P-2000, Sutter Instruments, from fire-polished 1.5 mm borosilicate glass and filled with pipette solution (PS, containing (in mM): K-gluconate (135), KCl (20), MgCl_2_ (2), HEPES (10), EGTA (0.1), Na_2_ATP (2), at pH = 7.3 adjusted with KOH). Pipette resistances were 5–10 MΩ. Measured liquid junction potential of 13 mV was not corrected. Control experiments were conducted with pipettes filled with BS. To estimate the origin of internal calcium elevations, neurons were pre-incubated in nominally 0 mM Ca^2+^ BS containing 2 mM EGTA, and the same solution was in the patch pipette. A Ag/AgCl reference electrode was used. Acquisition was via HEKA EPC10 dual USB patch-clamp amplifier (Heka Electronics GmbH) coupled to PatchMaster v2.901 software. Signal was sampled at 10–20 kHz, and low-pass filtered at 10 kHz (filter 1) and 3 kHz (filter 2). The EPC10 triggered imaging 10 ms after the start of recording, and recorded when images were acquired. Giga-seal was established after the pipette was positioned several µm above the target cell via Luigs & Neumann SM I micromanipulator. Relative pipette locations preceding the combined measurements were read out from a Luigs & Neumann SM IV focus control.

### Paired electrophysiology measurements

Dual and double patch-clamp measurements during imaging consisted of successive patching of the same and neighboring neurons, respectively, to detect the membrane responses while the second probe contacted the same cell or a neighboring neuron. Synaptic connections were validated by the presence of post-synaptic potentials or currents time-locked to the evoked APs in the neighbor. Post-synaptic deflections with $\ge$ 0.1 mV amplitude and $\le$ 10 ms latency were considered monosynaptic. The uncoupled pair was considered to share a common input if near-simultaneous post-synaptic potentials were detected. For double patch confirmation of the electrical coupling, measurements were done in the synaptic blocker cocktail described below.

Chips contained an 8 × 8 grid of 10 µm diameter opening Pt electrodes with 200 µm pitch. Two electrode designs were used: nanocavity and nanostraw-nanocavity electrodes. Fabrication and acquisition details can be found in^64^for nanocavities, and^65^ for nanostraws. The MEA signal was acquired via home-built headstage and amplifier, with total 101–1010 gain. The signal was sampled at 10 kHz, digitized in + /− 10 V, high-pass filtered at 72 Hz and low-pass filtered at 7.7 and 8.2 kHz.

### Imaging cytoskeleton

All chemicals were purchased from Sigma Aldrich, except Nuc Blue Fixed Stain (Invitrogen). The cells were fixed with 4% paraformaldehyde for 20 min (RT). Cytoskeleton buffer (CB), containing (in mM): EGTA (5), glucose (5), MES (10), MgCl_2_ (5), NaCl (150), streptomycin (1.72) was used for washing, permeabilization and staining. Samples were first washed by RT CB containing 30 mM glycine, and then 3 × with cold CB. The samples were permeabilized by 0.1% Triton X for 10 min and blocked with 5% milk powder for 1 h at RT. Following 3 × washing in cold CB, samples were incubated for 1 h with Phalloidin-488 (1:500) at RT. Samples were stained with Nuc Blue Fixed Stain for 10 min, washed 3 × in cold CB and stored at 4 °C in darkness. An Airy scan detector in “Resolution versus Sensitivity” mode at an LSM 880 (Zeiss) equipped with a Plan-Apochromat 63x/1.4 Oil DIC M27 objective (Zeiss) was used for imaging.

### Pharmacology

Compounds used in pharmacological investigations are described in Supplementary Table S1. Unless otherwise stated, chemicals were prepared in water and neurons were treated by bath application 5–10 min before the start of the measurement. Incubations > 30 min were done in a drug-containing NB medium at 37 °C.

### Neurobiotin injection

Electrically coupled neurons were detected by neurobiotin (ThermoFisher) injection followed by visualization through streptavidin-Alexa 488 (ThermoFisher). Neurons were patched with pipettes containing 10% neurobiotin, and filled iontophoretically, through 0.5 s 200 pA current pulses, at 1 Hz for 5–10 min^66^. Following the injection, neurons were incubated at 37 °C for ≥ 1.5 h to allow diffusion through neurites. The samples were fixed overnight in 4% paraformaldehyde at 4 °C. Cells were treated for 1–2 h at RT with permeabilization, blocking and staining solution containing 0.25% Triton-X, 2% BSA, and streptavidin-Alexa 488 (1:300)^67^. Samples were visualized on the AxioScope described above, using BP 450–90 nm excitation and LP 515 nm emission filters, using 2 × 2 binning.

### Experimental set-up and statistics

To test the neurons' electrophysiological properties after the neighbor’s mechanical deformation, dependent sampling was performed to avoid sampling bias^68^, and before/after conditions were processed the same. Due to the nature of single-neuron membrane deformation, independent datasets were collected for pharmacological investigations of mechanical responses to avoid any confounding effects from pipette movement. Neurons were pooled from E18 siblings of both sex, and control and treated populations of neurons were randomly assigned. A semi-automated analysis was performed the same on control and treated groups. Investigators weren’t blind to the pharmacological interventions during the analysis. Mechanical stimulation measurements were excluded when the membrane was injured during the pipette-membrane engagement, indicated by responses to the voltage test-pulses. Besides the pre-mature intracellular access, patch-clamp recordings were also excluded if a negative current greater than 150 pA was necessary to maintain the resting potential at − 60 mV. Multiple comparisons were corrected through Bonferroni corrections. Shapiro–Wilk test was used for normality. Parametric tests were used for normally distributed data, shown as mean ± SEM. Otherwise, non-parametric tests were used and data is shown as median and IQR. Statistical significance was evaluated at *p* ≤ 0.05. Data was not outlier-filtered or otherwise transformed. Box plots have boxes from the first to the third quartile, the median as a horizontal line, the mean as an x, and whiskers spanning over a 1.5 × inter-quartile range (IQR). Symbols indicating statistical significance within the figures are: ns: 0.05 < *p* ≤ 1.00, *: 0.01 < *p* ≤ 0.05, **: 0.001 < *p* ≤ 0.01, ***: 0.0001 < *p* ≤ 0.001, ****: *p* ≤ 0.0001.

### Supplementary Information

Supplementary Information.

## Data Availability

The datasets generated during and/or analyzed during the current study are available from the corresponding author on reasonable request.
