# Supplementary material for: Single-neuron mechanical perturbation evokes calcium plateaus that excite and modulate the network
Source: Sci Rep. 2023 Nov 24;13:20669. doi: 10.1038/s41598-023-47090-z (PMC10673841; doi:10.1038/s41598-023-47090-z)
Supplement: Supplementary file 1 — Supplementary Information. [file 41598_2023_47090_MOESM1_ESM.docx]

Supplementary

Single-neuron mechanical perturbation evokes calcium plateaus that excite and modulate the network

Bogdana Cepkenovic, Florian Friedland, Erik Noetzel, Vanessa Maybeck, Andreas Offenhäusser

1. Mechanical stimulation

2.Experimental estimation of shear stress

Figure S1. Diffusion of pipette constituents isn’t involved in calcium plateau generation

Figure S2. Giga-seal formation doesn’t induce membrane poration

Figure S3. The extent of pipette flow visualized by 0.01 % FITC staining of pipette solution

Figure S4. Calcium plateau propagation through the neurites to the soma of the neighbor

Figure S5. Physical connections mediate plateau propagation

Figure S6. Calcium plateaus extent of propagation in 3mm^2^ area

Figure S7. Single-neuron mechanical perturbation evokes network bursts

Figure S8. Depolarization kinetics of plateauing neurons

Figure S9. Factors defining depolarization kinetics and firing outcomes

Figure S10. Non-responding neurons contribute to major network bursts

Figure S11. TTX reliably prevents the generation of action potentials

Figure S12. Effects of pharmacological treatments on plateau amplitude

Figure S13. Bi-directional responsiveness during a conseuctive stimulation

**1. Mechanical stimulation via the pipette**

Preceeding the giga-seal, overpressure of +60-80 mmHg was syringe-applied and monitored via a manometer CE 0483, (error range: ±3 mmHg, BioPlus). Relative timings of the touch were *post-hoc* estimated from the electrical responses to the 5 mV, 5 ms, test pulses. The initial engagement of the pipette tip and the membrane is marked by a transient fluctuation of the baseline, and membrane indentation from the positive pressure (Fig. 1A approach and 1B red arrow). The slow advancement of the pipette is marked by a 0.1-0.6 MΩ increase, indicative of close contact, followed by the pressure release (Fig. 1A touch and 1B blue arrow) and weak suction to establish giga-seal. On average, the pipette engaged with the membrane at 9.2 ± 1.3 s, the pressure was released at 17.0 ± 0.8 s, and, if formed during the recording, giga-seal was at 46.2 ± 3.1 s from the start of the video (average ± SEM).

**2. Experimental estimation of shear stress**

We experimentally estimated the extent of the shear stress applied to the membrane due to liquid flow exiting the pipette tip. Pipette flow was estimated by mass loss during a 45 min +80 mmHg overpressure applied to the pipette in the bath. Pipette resistance and pressure were constantly monitored to prevent pipette tip clogging or pressure loss. Assuming the water density γ = 997 kg/m³, the pipette flow (G) was estimated as:

G = $\text{Δm}/{\text{Δt}\text{ }\text{*}\text{ }\text{γ }}\text{ =}$0.74 nL/s. (1)

Immediate exit velocity (v) is inversely related to the cross-section area of the pipette tip, resulting in

$\text{v =G/A=23.56 cm/s}$. (2)

Based on simulations of pipette flow from the work of Korchev et al,[1] maximum shear stress is proportional to the maximum velocity gradient, which is at one pipette radius above the surface. Assuming the water viscosity η = 1 mPa*s, the maximum shear stress was calculated as:

$\frac{\text{dv}}{\text{dz}}\text{* η = 230 pN/µm}\text{2}$. (3)

Since the mechanical responses further develop as the pipette approaches the cell, the calculated force is the lower boundary.

Table S1. Chemicals used to identify the origin and propagation mechanisms of calcium plateaus.

| **Compound (supplier)** | **Final concentration [µM]** | **Incubation time** |
| --- | --- | --- |
| GdCl_3_ (Sigma-Aldrich) | 50 | 5-10 min |
| Cytochalasin D (Sigma-Aldrich) | 10 (0.1%)* | 5h |
| Blebbistatin (-) (Sigma-Aldrich) | 50 | $\text{≥}$30 min |
| CdCl_2_ (Sigma-Aldrich) | 200 | 5-10 min |
| 2-APB (HelloBio) | 100 (0.1%)* | $\text{≥}$30 min |
| Mibefradil (Sigma-Aldrich) | 5 | 5-10 min |
| Nifedipine (Sigma-Aldrich) | 10 (0.05%)* | 5-10 min |
| TTX (Tocris) | 1 | 5-10 min |
| NBQX (Tocris) | 20 | 5-10 min |
| Bicuculine (Tocris) | 20 | 5-10 min |
| D-AP5 (Sigma-Aldrich) | 80 | 5-10 min |
| CBX (Tocris) | 100 | $\text{≥}$30 min |
| Mefloquine-hydrochloride  (Sigma-Aldrich) | 25-50 (0.05-0.1%)* | $\text{≥}$30 min |

*- dissolved in DMSO (v/v %)


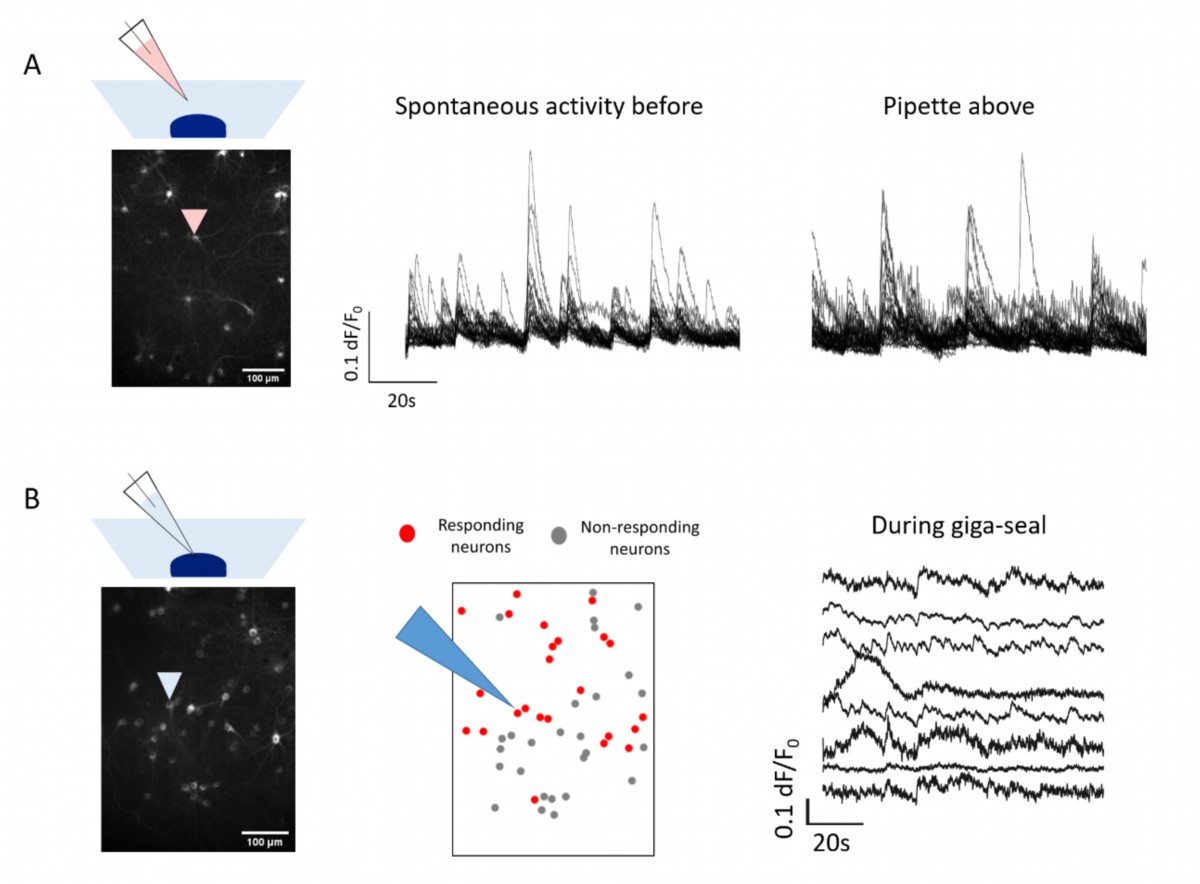


**Figure S1. Diffusion of pipette constituents does not generate calcium plateaus.** A: Pipette containing internal patch solution under overpressure, placed 5um above the marked cell, didn’t evoke calcium plateaus. From spontaneous activity traces before (middle) and during the diffusion of the pipette solution (right), it could be appreciated that only highly-correlated, AP-associated Ca^2+^ spikes are present. B: Patching the neuron (blue triangle) with bath solution containing pipette evoked calcium plateaus in the target and the surrounding neurons (red).

Current responses to 5mV-5ms voltage pulses are indicative if the pipette penetrates the cell. During the giga-seal formation, premature slow capacitive transients similar to the membrane opening in whole-cell mark intracellular access due to membrane injury. Corresponding calcium trace shows a strong internal calcium increase due to ion leakage around the injury site/extensive Ca^2+^ mobilization from injured internal depots. Only recordings without the membrane injury were considered. Calcium plateaus could arise due to membrane poration caused by shear stress from the liquid flow. Membrane poration could have led to the membrane resistance drop due to leakage currents, unseen by the test pulses of the approaching pipette. To test if the shear stress from the liquid flow induced smaller pores at the membrane, we performed giga-seal acquisition in trypan-blue (Fig. 1F). Here (Fig. S2B), we show that in control with ruptured membrane, a weak staining via trypan blue could be detected.

**
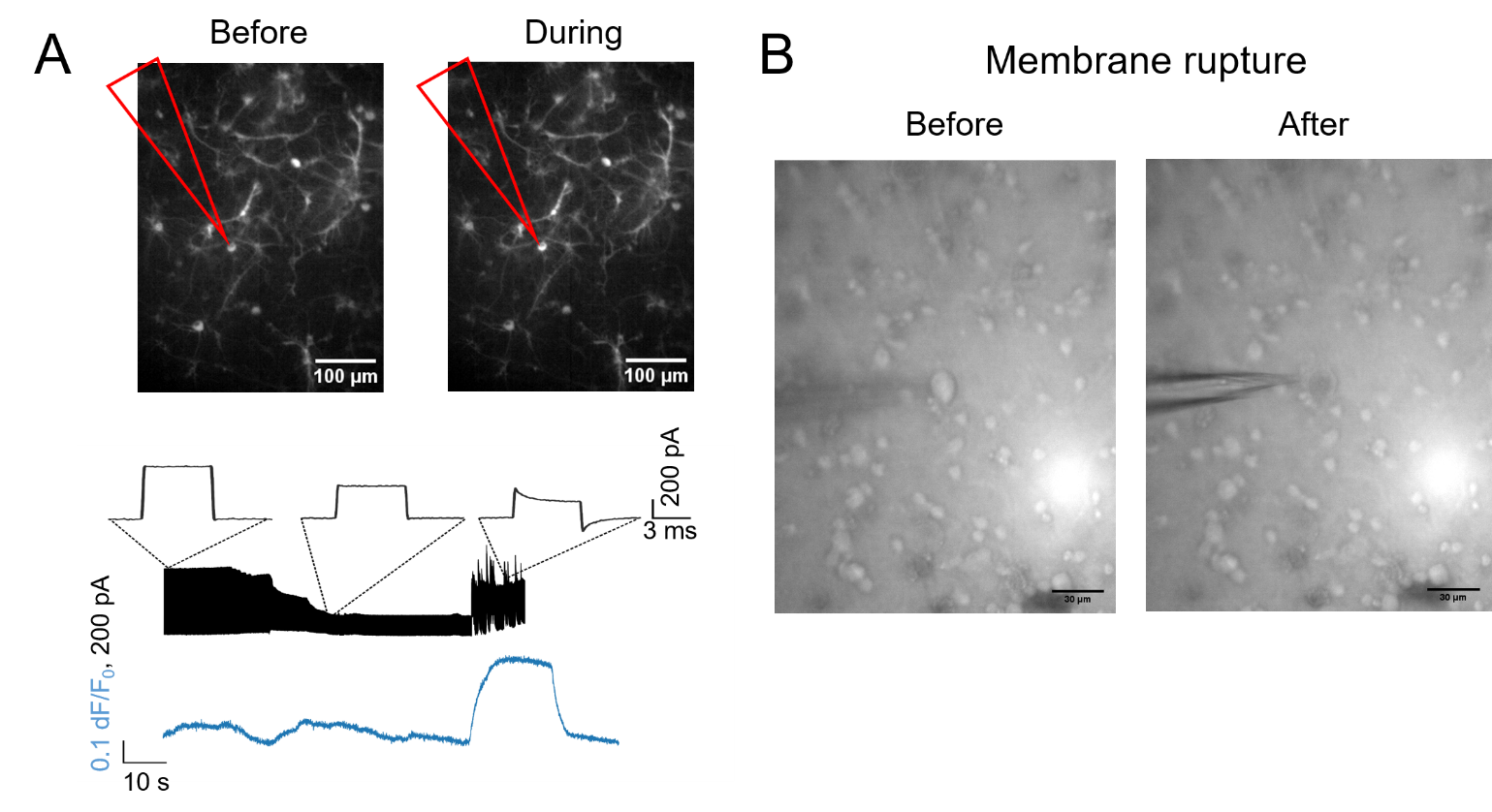
**

**Figure S2. Giga-seal formation doesn’t induce membrane poration.** A. Current responses to pipette test pulses are indicative of membrane poration preceding the giga-seal establishment. Top: jRCaMP signal before and during the neuron’s (red triangle) membrane poration mediated by the pipette. Bottom: Simultaneous pipette responses to the 5 mV, 5 ms test pulses (black) and calcium trace of the target neuron (blue). The initial contact point is followed by a slight current drop due to the increase in the pipette resistance. Concurrently with the adverse penetration during the giga-seal establishment, current responses gain slow capacitive transients indicative of intracellular access. B. Positive control for trypan-blue assays shows the previously transparent neuron (left, pipette above out of focus) turns dark (right) following the membrane rupture.

**
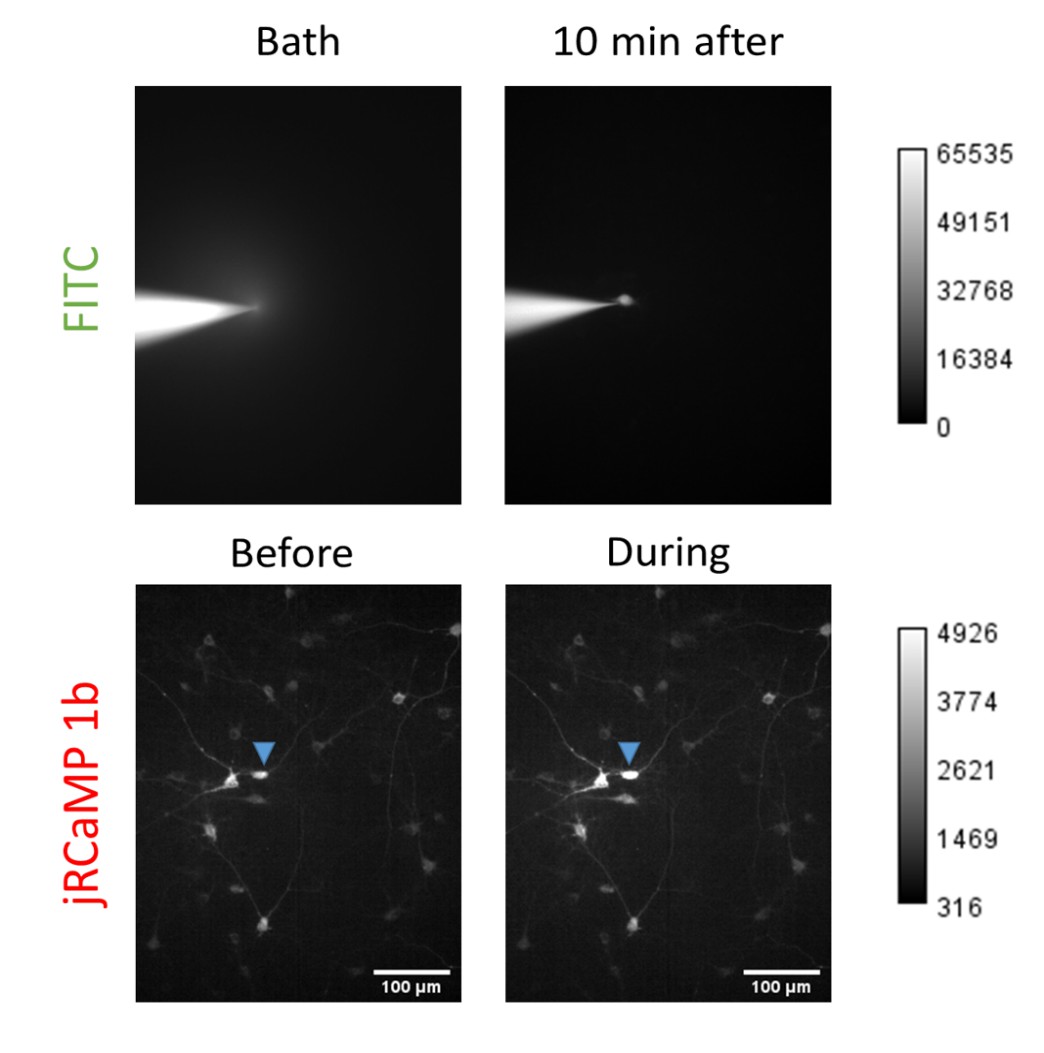
**

**Figure S3. Extent of flow vs. extent of calcium plateaus.** Top: The range of flow-related effects visualized with FITC. Top left: under 80 mmHg overpressure, with pipette above the cell layer. Top right: FITC signal captured post-giga-seal. The right calibration curve depicts the pixel intensity of both top micrographs. Bottom: Extent of mechanically evoked calcium responses within the same region. Bottom left: a snapshot of the local network with a pipette localized above the target neuron (blue arrow). Bottom right: jRCaMP micrographs of the same region during the giga-seal configuration. The right calibration bar depicts the pixel intensities of both bottom micrographs. Bottom scale bars are universal to bottom and top micrographs.


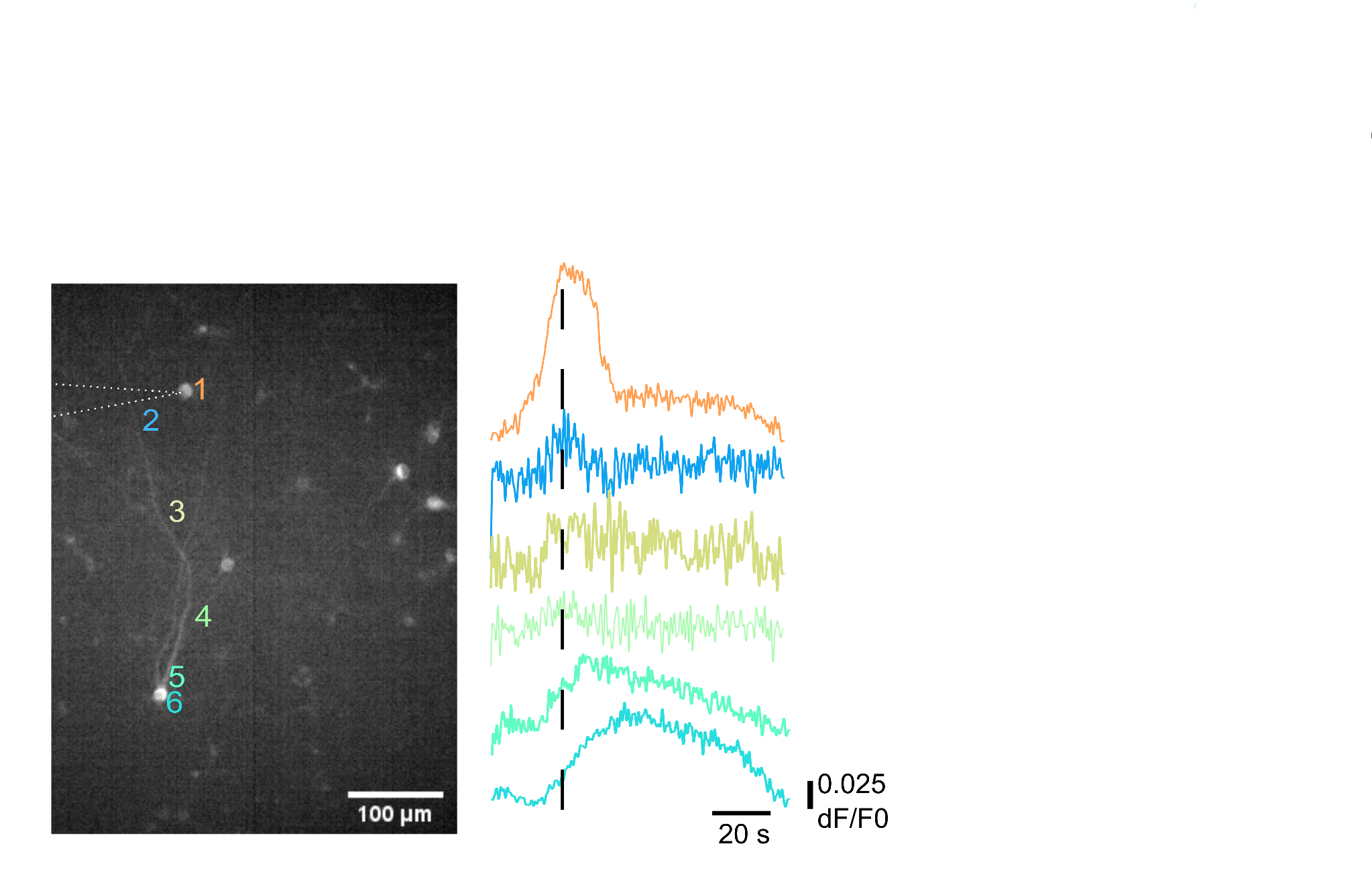


**Figure S4. Calcium plateau propagation from the soma of the target neuron, through the neurite and the soma of the neighbor.** Left: jRCaMP signal to depict the position of the target neuron. Numbers mark the positions of ROI positions where calcium traces on the right were extracted. Calcium traces are color-coded to match the ROI numbers on the left. Vertical line marks the local maximum of plateau initiated at a target soma (1).


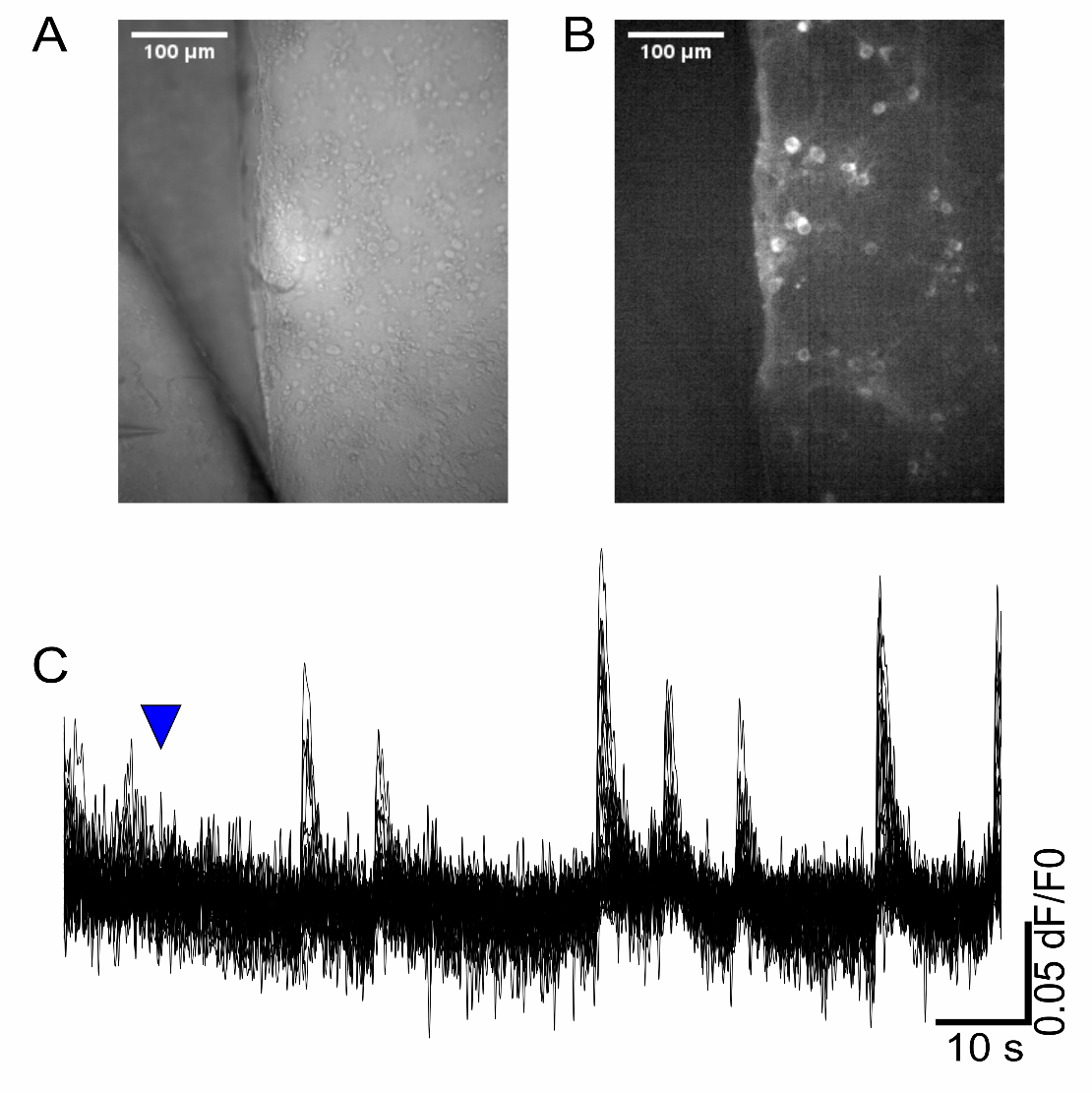


**Figure S5. Physical connections mediate plateau propagation.** A. A brightfield snapshot of a two-coverslip experiment used to determine the mechanism of calcium plateau generation. Coverslips were in different focal planes, and the micrograph represents the superposition of the brightfield snapshots with a focus on the left and the right coverslip. The left coverslip contained the mechanically stimulated neuron, while the calcium imaging was focused on the right coverslip. B. JRCaMP signal of the right coverslip. C. Calcium traces during the mechanical stimulation of the neuron on the left coverslip in A. Timing of the mechanical stimulus marked by a blue arrow.


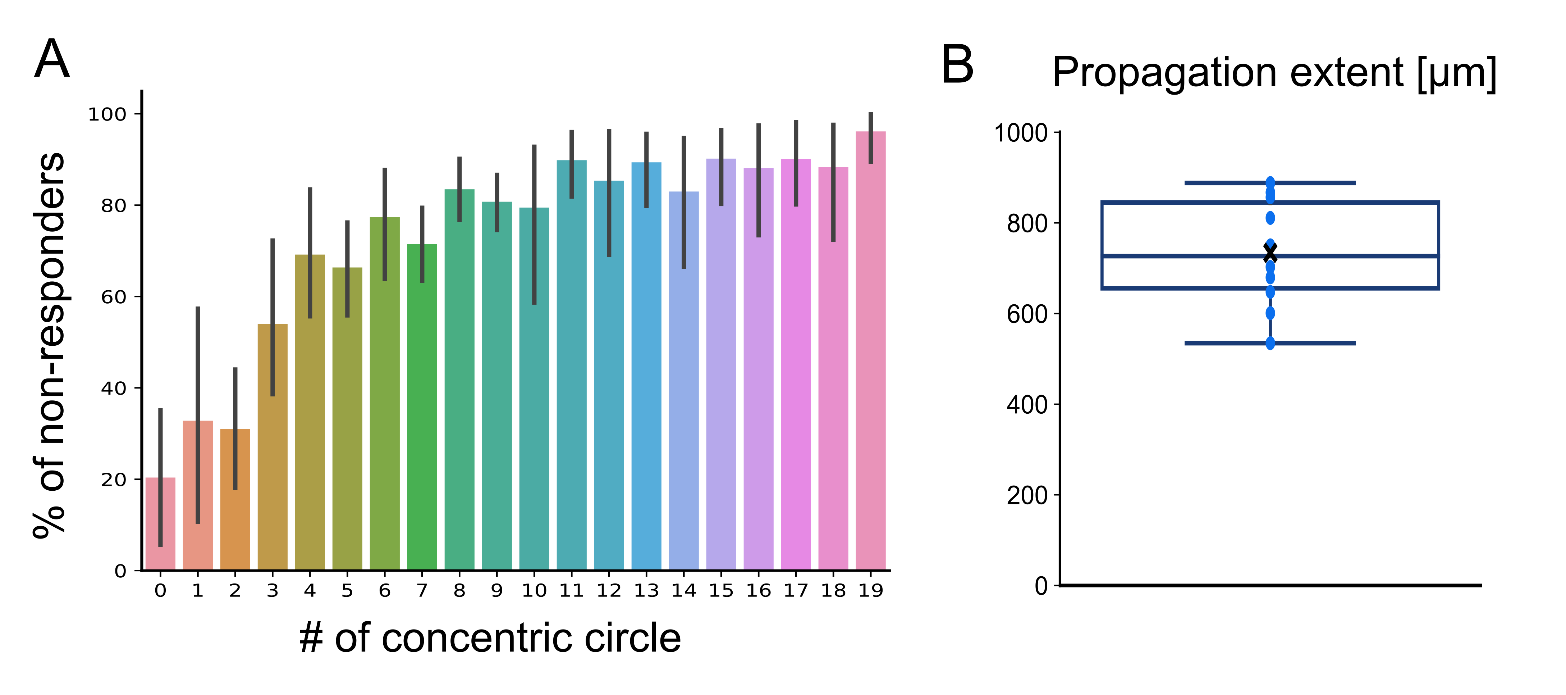


**Figure S6. Calcium plateaus extent of propagation in 3mm^2^ area.**A. % of non-responders around the target, in concentric circle with radius ~46µm x circle number. Bar plots represent the mean for 10 fields of view with whiskers corresponding to 95% CI. The fraction of non-responders rose to 90% at about 450 µm from the target. B. Maximum propagation extents are depicted in a box plot and a swarm plot.


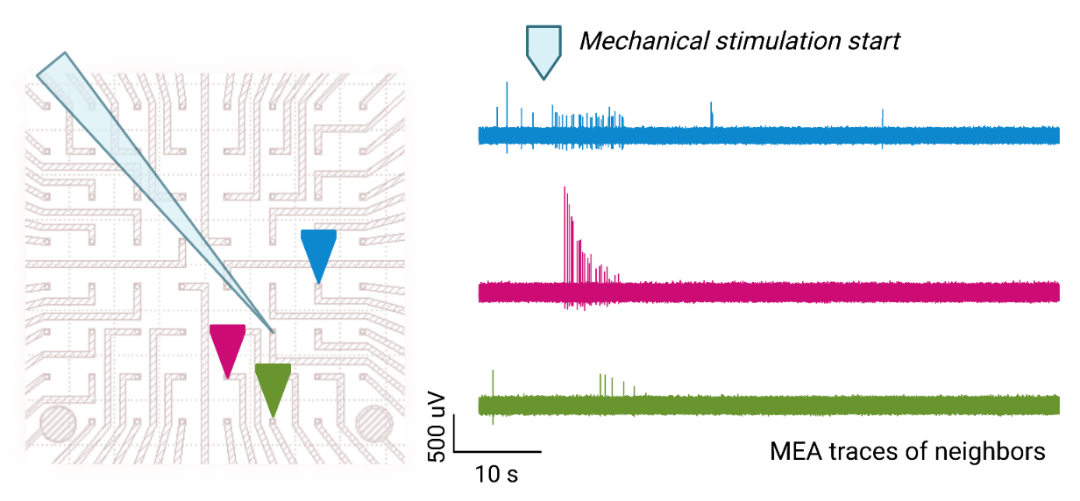


**Figure S7. Single-neuron mechanical perturbation evokes network bursts.** MEA recordings during the single-neuron mechanical stimulation reveal bursting activity in electrodes a few hundred microns away. Left: schematic of on-chip patch-clamp mechanical stimulation of a neuron near the electrode marked by pipette. Right: MEA traces of responding electrodes, color-coded to match the positions on the left.

Plateauing neurons consistently showed slow membrane depolarizations, and only sporadically were APs evoked via the mechanical stimulus (Fig. 4). Figure S8A shows intracellular traces of bursting and non-bursting plateauing neurons. To estimate the kinetics of slow depolarization, signal was filtered by a Savitzky-Golay filter with a 0.625 s long window and third polynomial order. These polynomial filter parameters allowed slow component extraction without signal deviation (red trace in Fig. S8A). The UP kinetics of the membrane depolarization were estimated from the maximum of first time-derivative of the baseline (blue trace in Fig. S8A). Boxplots in Fig. S8B demonstrate that the bursting occurs in cells with faster UP kinetics and stronger depolarization.


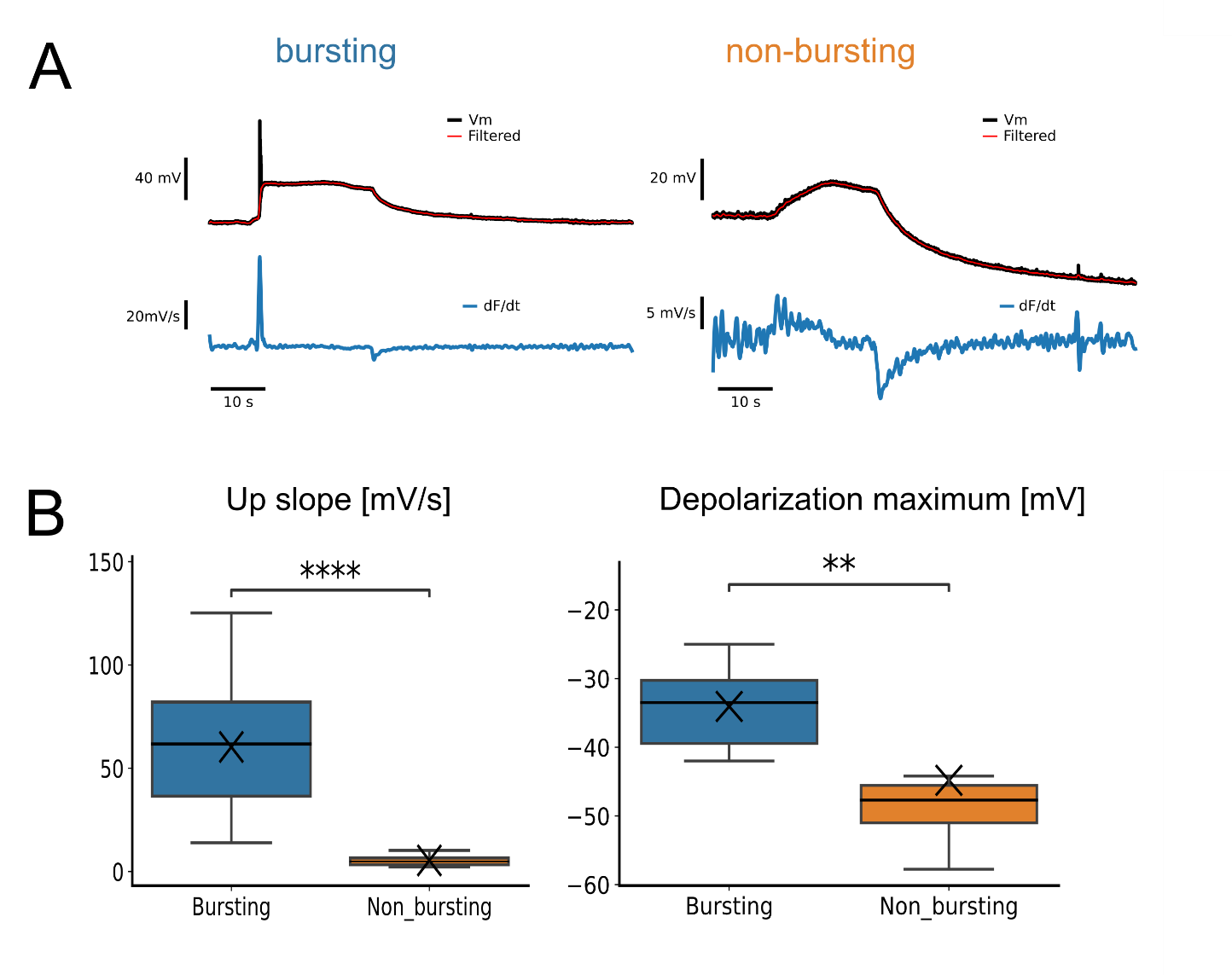


**Figure S8. Depolarization kinetics of plateauing neurons.** A. Differences in depolarization kinetics among the neurons with bursting or plateaus only. Membrane potential (Vm) was filtered to obtain the baseline (red) cleared of APs. The temporal first derivative of the baseline (blue) was used to estimate the maximum of the UP slope of slow mechanoresponses. B. Boxplots depicting the UP slope distribution (left) and depolarization maxima (right) among bursting (N=10) and non-bursting neighbors (N=12).

The eventual inter-trial differences in mechanical stimulation (Fig. 1) were indirectly tested by focusing on the responses of the target cell. The firing outcome of the neighbor responding to a nearby neuron’s stimulation wasn’t dependent on the amplitude (Fig. S9B) or the slope (Fig. S9A) of calcium plateaus developed at the target. The distance from the target as well didn’t indicate the responder’s firing outcome (Fig. S9C), supporting that external factors don’t determine depolarization kinetics. Membrane time constant (Fig S9D) was similar across the plateau only and bursting responders.


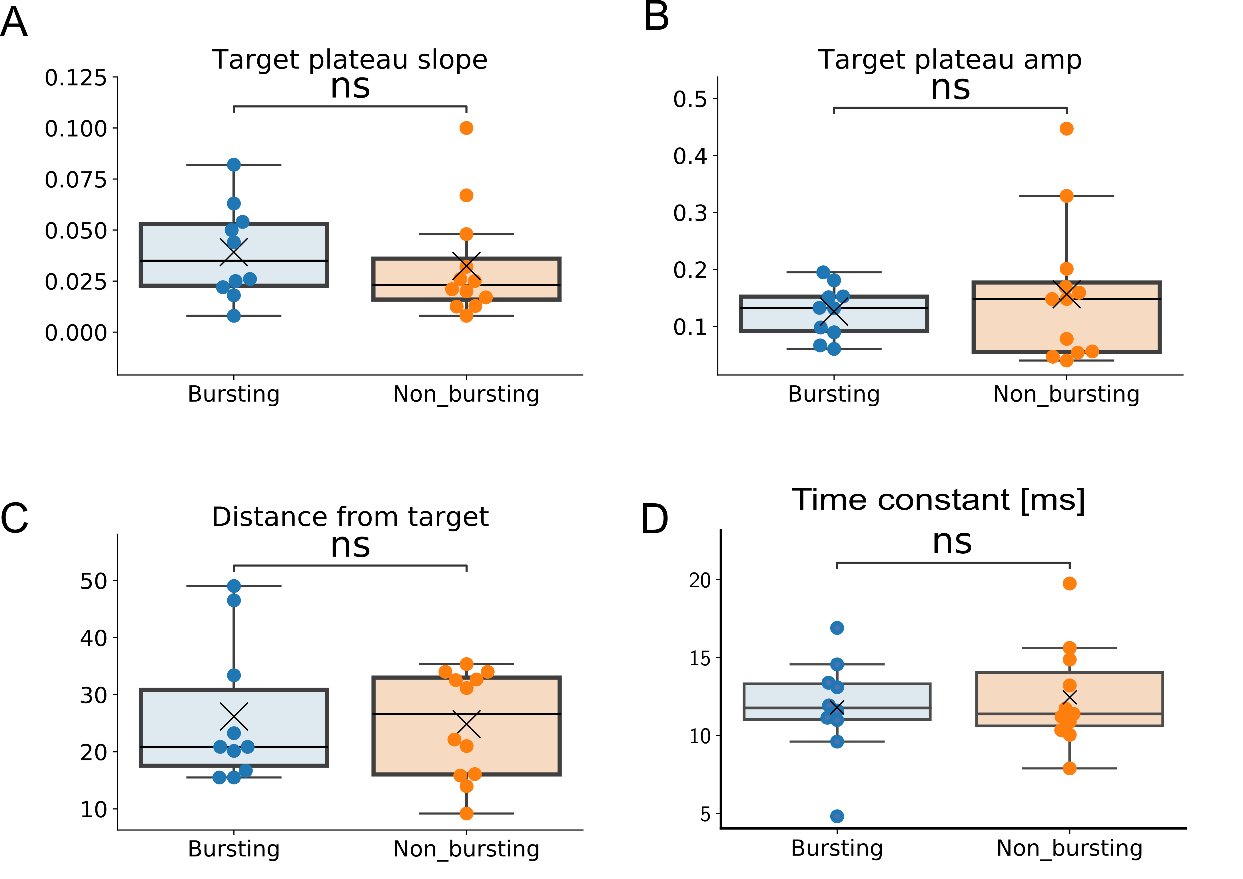


**Figure S9.** **Factors defining depolarization kinetics and firing outcomes**. A. Comparison of the calcium plateau UP slope at the initiation site. B. Calcium plateau amplitude at the target. C. Distance of plateauing neuron from the stimulated neuron. D. Membrane’s time constant extracted from the exponential fit of voltage response to -20 pA current pulse.

Spontaneous network activity was sampled prior to mechanical deformation via calcium imaging to evaluate functional connections from correlations. As previously reported, in vitro networks engaged in bursts during which many neurons fired synchronously. The main network bursts were defined from the convolved sum of calcium peaks, with the soft threshold of mean + 5*standard deviations. Neurons were considered as burst-synchronized if their spiking consistently fell within 2 s window around the major network bursts (MNB). While there was a significantly higher fraction of MNB participating neurons that responded to the target neuron deformation (63%), about 53% of non-responders also participated in MNBs, confirming that network-wide synchronizations may overwrite functional connections estimated at the local network level.


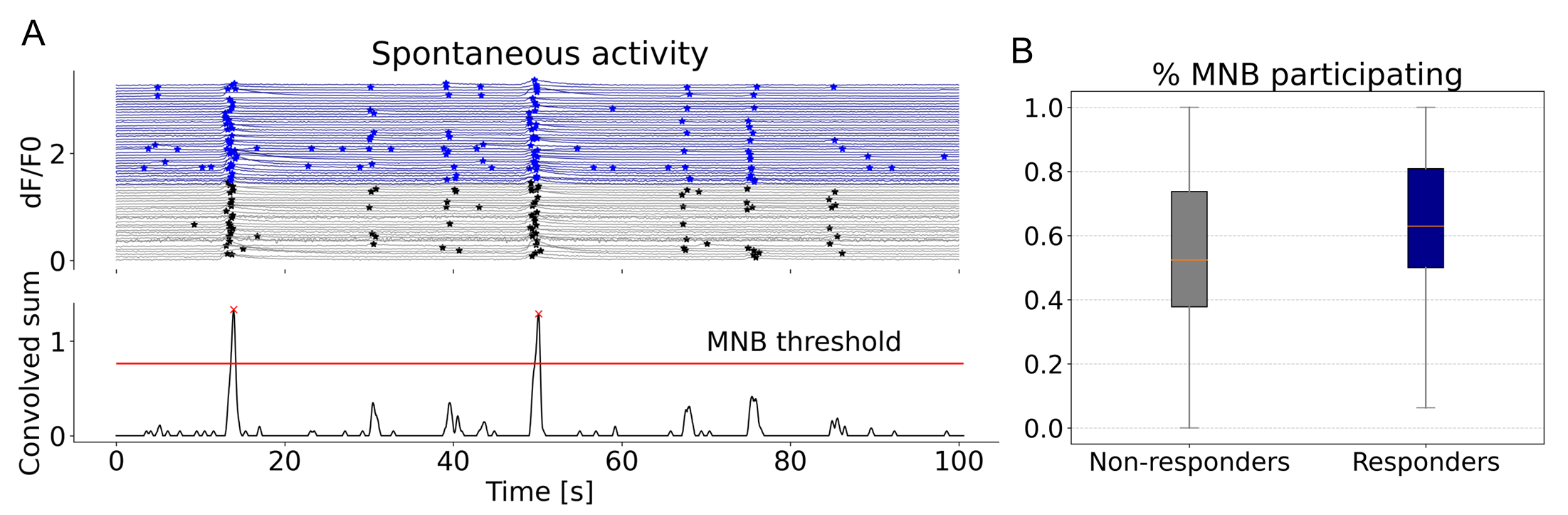


**Figure S10. Non-responding neurons contribute to major network bursts.** A. A 100 s network activity measurement via calcium imaging, depicting calcium traces and peaks (*-ed) of responding (blue) and non-responding neurons (gray). Black trace denotes convolved sum of binarized calcium traces and definition of major network bursts by soft threshold. B. Fractions of MNB participating neurons among non-responders and responders.


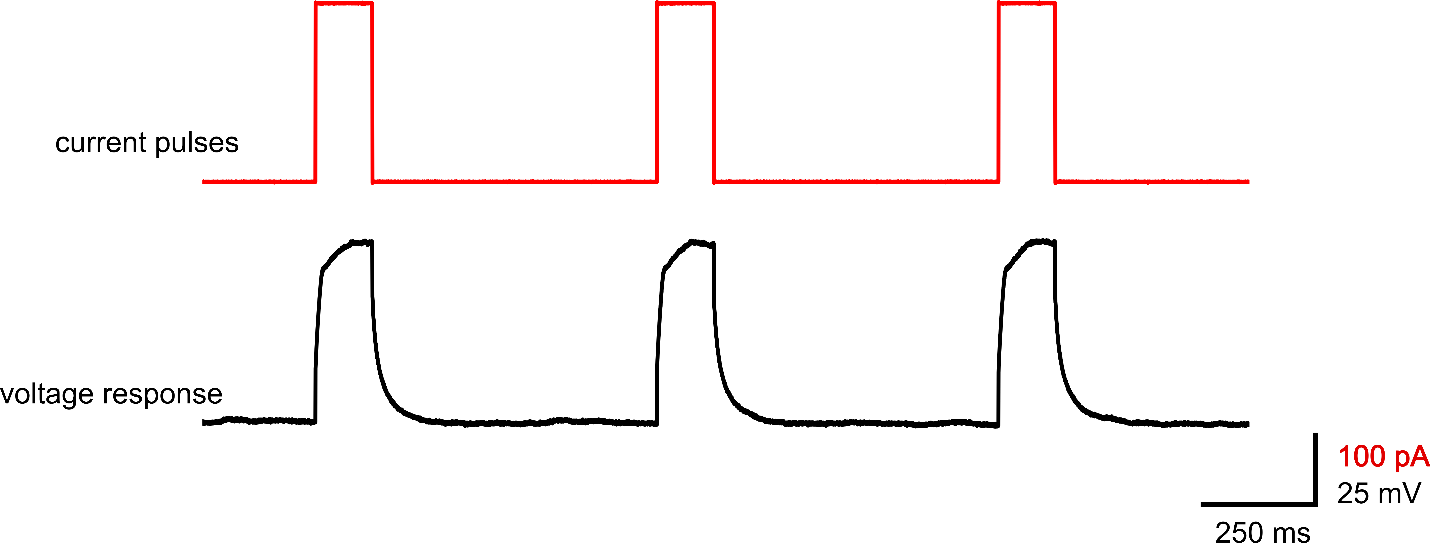


**Figure S11. TTX treatment efficiently blocked the generation of action potentials.** Top trace: 250 pA depolarizing current pulses applied via the patch-pipette in whole-cell current clamp mode. Bottom trace: voltage responses to the top current pulses; showing suprathreshold depolarizations without APs.


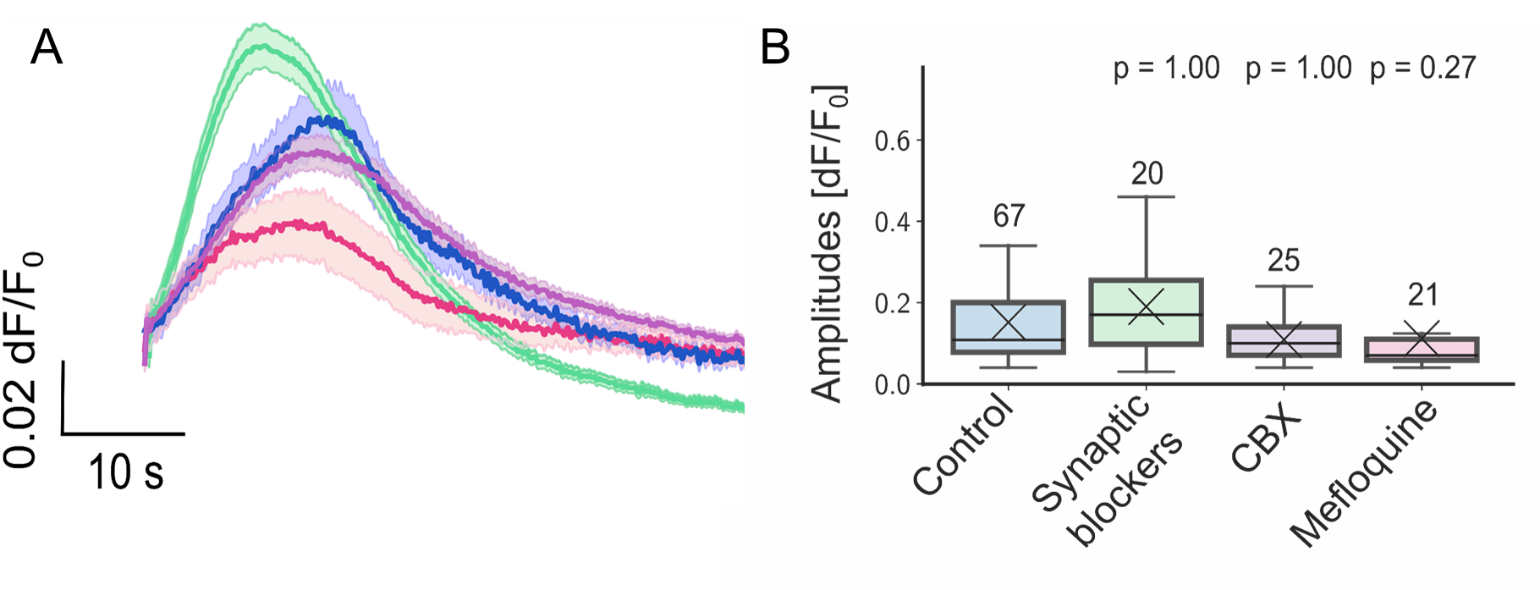


**Figure S12. Effects of pharmacological treatments on calcium plateau amplitude.**A. Calcium plateau amplitudes in the treated and untreated group. Mean plateaus aligned to onsets with a shaded area corresponding to SEM. The color code matches the box plots. B. Plateau amplitude distribution in control, synaptic, and gap junction blockers. The numbers above whiskers indicate the sample size, top: p-values from the two-tailed Mann-Whitney test with Bonferroni correction.


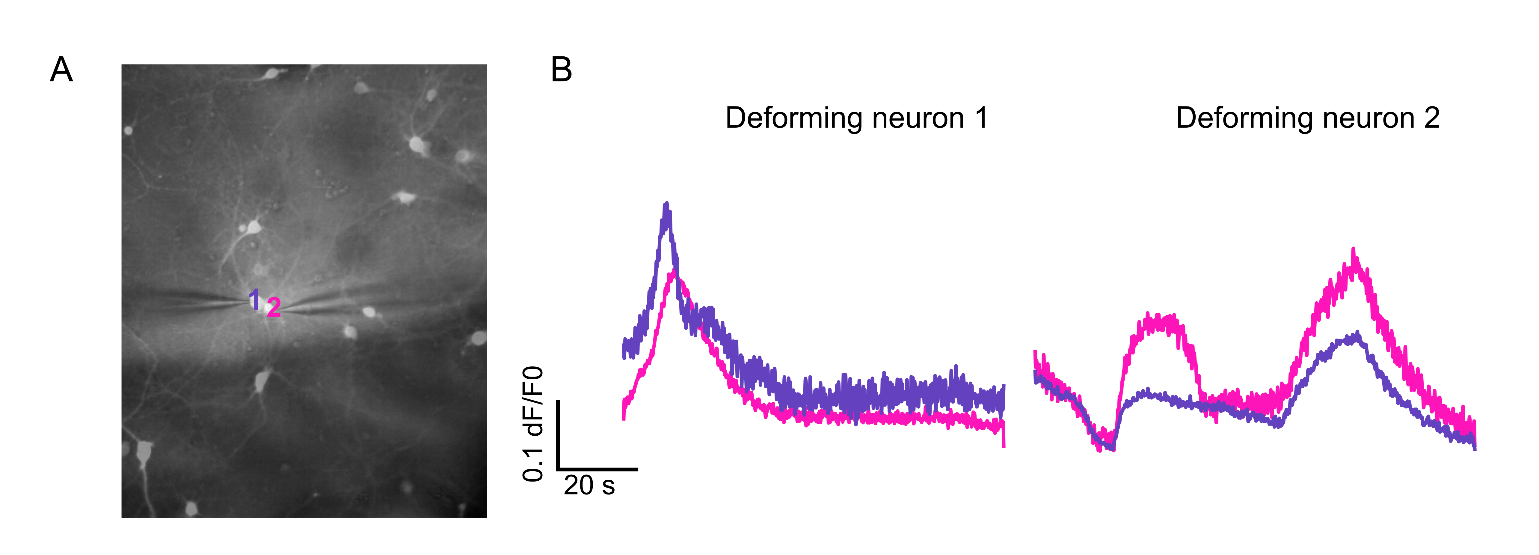


**Figure S13. Bi-directional responsiveness during a consecutive mechanostimulation.**Successive stimulation of 1 neuron evoked calcium plateaus in the neighbor and vice versa.

1. Sánchez, D.; Johnson *et. al*. Noncontact measurement of the local mechanical properties of living cells using pressure applied via a pipette. *Biophys J* . **95**, 3017-27. (2008).
